# Conditional control of RNA-guided nucleic acid cleavage and gene editing

**DOI:** 10.1038/s41467-019-13765-3

**Published:** 2020-01-03

**Authors:** Shao-Ru Wang, Ling-Yu Wu, Hai-Yan Huang, Wei Xiong, Jian Liu, Lai Wei, Ping Yin, Tian Tian, Xiang Zhou

**Affiliations:** 1 College of Chemistry and Molecular Sciences, Key Laboratory of Biomedical Polymers of Ministry of Education, Hubei Province Key Laboratory of Allergy and Immunology, Wuhan University, 430072 Wuhan, China; 2 Sauvage Center for Molecular Sciences, Wuhan University, 430072 Wuhan, China; 3 National Key Laboratory of Crop Genetic Improvement and National Center of Plant Gene Research, Huazhong Agricultural University, 430070 Wuhan, China

**Keywords:** Nucleic acids, CRISPR-Cas9 genome editing

## Abstract

Prokaryotes use repetitive genomic elements termed CRISPR (clustered regularly interspaced short palindromic repeats) to destroy invading genetic molecules. Although CRISPR systems have been widely used in DNA and RNA technology, certain adverse effects do occur. For example, constitutively active CRISPR systems may lead to a certain risk of off-target effects. Here, we introduce post-synthetic masking and chemical activation of guide RNA (gRNA) to controlling CRISPR systems. An RNA structure profiling probe (2-azidomethylnicotinic acid imidazolide) is used. Moreover, we accomplish conditional control of gene editing in live cells. This proof-of-concept study demonstrates promising potential of chemical activation of gRNAs as a versatile tool for chemical biology.

## Abstract (web summary)

Constituitively active CRISPR systems have the risk of adverse off-target effects. Here the authors use chemical masking and activation of gRNA to control activity.

## Introduction

Clustered regularly interspaced short palindromic repeats (CRISPR) represents a prokaryotic adaptive immune system that provides acquired resistance against invading genetic molecules^1–4^. The artificial CRISPR systems are a highly diverse group, which usually consists of two important components, CRISPR associated protein (Cas) and guide RNA (gRNA)^5–7^. To date, CRISPR/Cas9 system has been heavily utilized as genome editing tools in cells^6,8^. In contrast to Cas9, Cas13a (also known as C2c2), has several unique properties that further expand the CRISPR toolboxes^9–11^. Despite the great promise of the CRISPR technology, uncontrolled CRISPR systems could have unpredictable off-target effects^12–14^. In this regard, some important methods have been developed for improving CRISPR specificity by using modified Cas9 proteins^15,16^ and modified gRNAs^17^. Another important way to mitigate off-target effects is by exerting external control over these systems^18–21^. In recent years, chemical regulation of proteins has been performed in living systems^22–24^. A significant progress has been made depending on light-induced protein dimerization^25^. Importantly, light-sensitive groups have been used to manipulate the function of Cas9 proteins in live cells with excellent specificity^26^. Although these optogenetic approaches have the advantage of spatially localized activation, it carries the limitation of requiring to perform genetic engineering and to protect light-labile protein during preparation and processing. Strikingly, targeted gRNA engineering to control CRISPR functions still remains to be explored.

The gRNA has been found to play essential roles in CRISPR systems, thus representing an attractive target to chemical manipulation^17,27–29^. In a previous study, Bhatia and co-workers have developed a robust light-activated CRISPR system^30^. However, to perform such regulation, additional protector DNA complementary to gRNA must be used. Recently, RNA engineering for chemical and biological applications has emerged as an exciting research field^31–33^. In a recent study, Kool and colleagues have developed a chemical strategy that achieved robust blockage of RNA structure by chemical acylation, and efficient recovery of RNA structure with a subsequent de-acylation procedure^34^. This strategy is promising and we envisioned that it will benefit the development of chemical switches to controlling CRISPR systems.

**Fig. 1:**
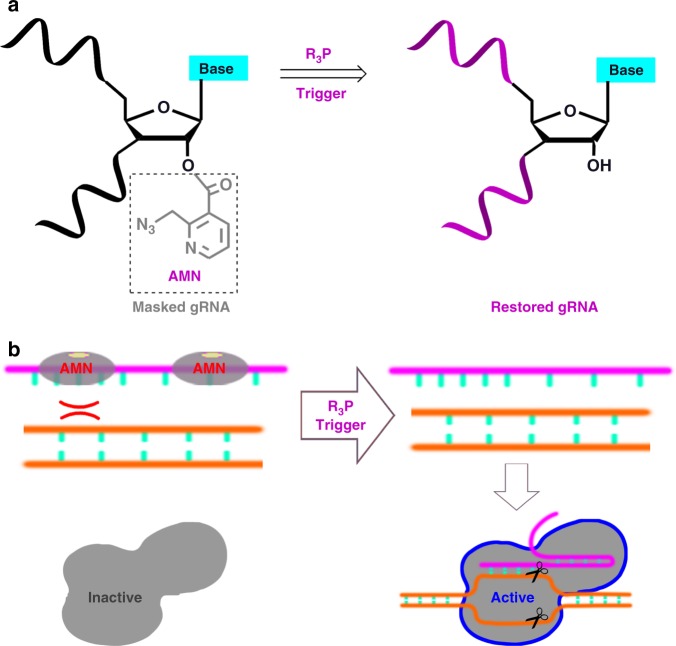
Schematic illustration of the design and workflow. **a** The post-synthetic masking was used to block gRNA biochemical activity, whereas the Staudinger reduction restored gRNA function. **b** Chemical activation of gRNA to controlling CRISPR systems.

In the present study, post-synthetic masking and chemical activation of gRNAs are developed to controlling CRISPR systems. An RNA structure profiling probe, 2-azidomethylnicotinic acid imidazolide (NAI-N_3_ in Supplementary Fig. 1), is used. Here, we show that covalent attachment of the azidomethylnicotinyl (AMN) groups efficiently blocks the function of gRNA and CRISPR systems (Supplementary Fig. 1). Importantly, with the action of Staudinger reduction, these systems restore their native activities (Fig. 1). This methodology functions both with CRISPR/Cas9 and CRISPR/Cas13a. Using various methods, we further demonstrate that, the AMN groups probably play roles by interfering the interactions among gRNA, Cas and target nucleic acid. In addition, chemically masked gRNAs are observed to be highly resistant to different Ribonucleases (RNases). Moreover, conditional control of gene editing in live cells is performed. This proof-of-concept study thus shows promising potential of chemical activation of gRNAs for conditional control of in vivo gene editing.

## Results

### The design of our strategy

The present study aimed to develop a strategy to control nucleic acid cleavage and gene editing in live cells. In CRISPR systems, the gRNAs are thought to play a wide range of catalytic or regulatory roles^35–37^. Our strategy was therefore designed to regulate the structure and function of gRNAs. Traditionally, the conjugation of chemical moiety to the oligonucleotides may interfere the access of other biomolecules^38^. Given ubiquitous 2′-OH groups in RNA ribose, the protection of such groups will constitute an initial step^34^. The gRNA function was expected to be temporarily suspended by attaching the AMN groups, which can be removed by treatment with phosphine through a Staudinger reduction^24,34^. Hence, we were interested in exploring how the post-synthetic masking and chemically selective unmasking of gRNA can be applied to reprogram CRISPR systems.

### Conditional control of RNA-guided DNA cleavage

**Fig. 2:**
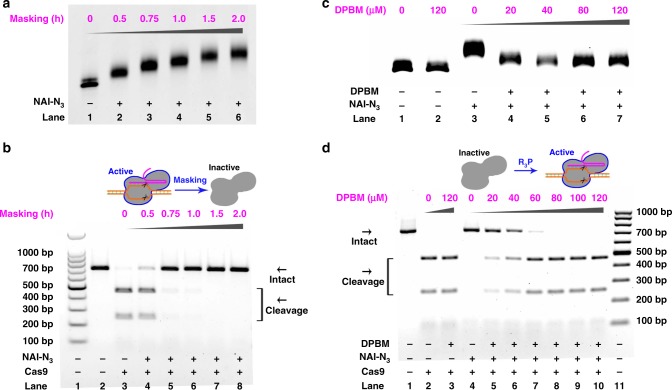
Conditional control of RNA-guided DNA cleavage. Reactions were carried out as described in the Experimental section. All samples were tested in three biological replicates. Image of representative data is shown here. Uncleaved t-GFP1 DNA (702 bp) cut to shorter cleavage fragments (469 bp and 233 bp) were indicated. **a**, **b** The influence of chemical masking of gRNA on Cas9 cleavage of t-GFP1. The gRNA (gRNA-GFP) was synthesized by in vitro transcription with T7 RNA polymerase. **c**, **d** The influence of DPBM on Cas9 cleavage of t-GFP1. The CRISPR/Cas9 system with acylated gRNA-GFP (200 mM NAI-N_3_, 2 h) was treated with various concentrations of DPBM. Source data is available in the Source Data file.

The Cas9 is an RNA-guided DNA endonuclease found in *Streptococcus pyogenes*, which can generate site-specific double-strand breaks based on the gRNA-defined sequence^5,35^. A gRNA targeting two different GFP (Green Fluorescent Protein) sequences was designed and synthesized by in vitro transcription with T7 RNA polymerase (gRNA-GFP in Supplementary Table 1 and Supplementary Fig. 2)^30^. An initial study was then performed to chemically mask this gRNA construct^34^. After quenching the reaction, the gRNA products were recovered by ethanol precipitation and subjected to denaturing electrophoresis. In this assay, the more slowly migrating bands represented the higher-molecular weight gRNAs with acylation (Fig. 2a). An important issue we next addressed was whether the acylation of gRNA can inhibit the function of CRISPR/Cas9. For proof of specificity, we performed an in vitro DNA cleavage assay targeting different GFP fragments (Schematic illustration in Supplementary Fig. 2). On the basis of our observations (lanes 4–8 in Fig. 2b and Supplementary Fig. 3), the acylation efficiently blocked site-specific DNA cleavage. We wished to determine the fraction of adducts of gRNA that were needed to inactivate the gRNA. In theory, the ribose 2′-OH acylation is accompanied by a 160 Da molecular weight (MW) increment corresponding to one AMN adduct, which means that the MW of two AMN adducts was roughly equal to the mean MW of one nucleobase. For a 1 h masking, up to eight AMN adducts were clearly recognizable by comparing the sample bands to the RNA markers (Supplementary Fig. 4). These results suggested that eight nucleobases need to be modified to inactivate a 102-nt gRNA. Since the guide sequences of a gRNA were approximately 20-nt in length, approximately two modifications were expected to occur in the guide sequences to inactivate the gRNA.

We anticipated that Staudinger reduction can drive the removal of AMN groups and reactivate CRISPR/Cas9 functions^24,34^. A variety of phosphines (each structure in Supplementary Fig. 5), including 2-(diphenylphosphanyl)benzamide (DPBM), 2-(diphenylphosphino)benzoic acid (DPBA), sodium diphenylphosphinobenzene-3-sulfonate (DPBS) and tris(2-carboxyethyl)phosphine hydrochloride (TCEP) have been examined^24,34^. The observations showed that all phosphines except TCEP cause efficient de-acylation at concentrations less than 120 μM (Fig. 2c and Supplementary Fig. 6). Additionally, each phosphine did not affect the movement of original gRNAs. We further tested whether Cas9 coupling with masked gRNA would show restored activity in response to phosphine treatments. Being consistent with the above de-acylation assay, we identified three phosphines (DPBM, DPBA, and DPBS) as being effective in recovery of site-specific DNA cleavage. Figure 2d and Supplementary Fig. 7 show the concentration-dependent recovery of Cas9 cleavage of different substrates with DPBM. In addition, the cleavage of target DNA with original gRNA was insensitive to DPBM concentration as high as 120 μM (lane 3 in Fig. 2d). The treatment with other phosphines (DPBA and DPBS) could also lead to substantial recovery of site-specific DNA cleavage as a consequence of chemical unmasking (Supplementary Figs. 8–10). On the basis of all these results, we ultimately identified DPBM as being optimal for further studies.

We wished to evaluate the pH effects on the Staudinger reduction. The CRISPR/Cas9 system was examined in the reaction buffer at different pH ranging from 5.9 to 8.9. Although the low pH value (pH = 5.9) resulted in a complete loss of activity for Cas9, the Staudinger reduction was insensitive to a change in the pH of the reaction medium. Our results clearly demonstrated that CRISPR/Cas9 can be regulated through Staudinger reduction in the pH range from 6.9 to 8.9 (Supplementary Fig. 11). We further addressed the possibility of spontaneous unmasking of gRNA adducts. For this purpose, 2-methylnicotinic acid imidazolide (mNAI), a closely related derivative of NAI-N_3_, has been synthesized as a negative control compound (details in Supplementary Methods). A long time-course experiment using individual phosphines alongside mNAI was performed (Supplementary Fig. 12). On the basis of our results, there was almost no spontaneous unmasking over 24 h, indicating that the gRNA adducts were stable under physiological conditions. Additionally, the acylated gRNA with mNAI exhibited high pH stability over a broad pH range of pH (Supplementary Fig. 13).

From a structural point of view, the gRNA is made up of two separate crispr RNA (crRNA) and tracrRNA. The crRNA is a 17–20 nucleotide sequence complementary to the target DNA, and the tracrRNA serves as a binding scaffold for the Cas9 nuclease. We wished to determine whether the current method can be applied to separate crRNAs and tracrRNAs. In this assay, DNA cleavage reactions were performed by individually masking the crRNA and tracrRNA components and then mixing/matching them with unmodified counterparts. On the basis of our results, the Cas9 activity was significantly blocked by masking of crRNA or tracrRNA (Supplementary Fig. 14). Moreover, activation of the complex was induced by treating with DBPM to release the blocking groups (Supplementary Fig. 15).

### Conditional control of RNA-guided RNA cleavage

**Fig. 3:**
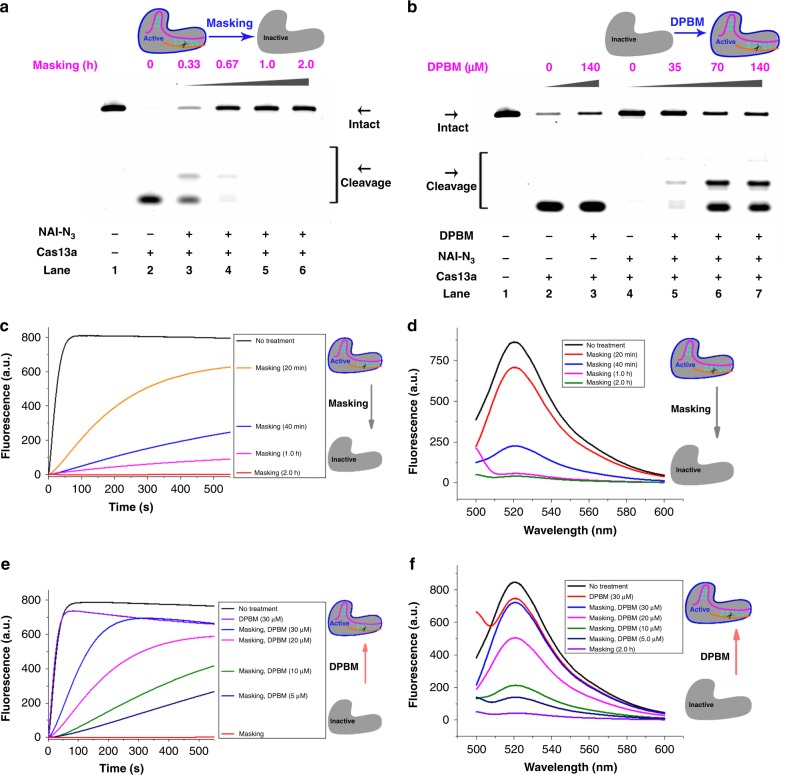
Conditional control of Cas13a cleavage. Reactions were carried out as described in the Experimental section. All samples were tested in three biological replicates. Image of representative data is shown here. The fluorescence of the sample versus time was shown. **a**, **c**, **e** The influence of chemical masking on Cas13a cleavage. The crRNA13a was masked with NAI-N_3_ (200 mM) for different durations. **b**, **d**, **f** The influence of DPBM on Cas13a cleavage. The CRISPR/Cas13a system with masked crRNA13a (200 mM NAI-N_3_, 2.0 h) was treated with various concentrations of DPBM. Source data is available in the Source Data file.

The Cas13a belongs to a type VI-A CRISPR RNase, which has been established as a useful tool for a variety of RNA targeting applications^10,39,40^. A prominent difference between Cas13a and Cas9 is that Cas13a binds and cleaves RNA rather than DNA substrates^36^. After achieving control of DNA cleavage, we further tested the effectiveness of our strategy for RNA cleavage control. The Cas13a from *Leptotrichia buccalis* (LbuCas13a) was selected for the proof of concept study^10^. Supplementary Fig. 16A clearly demonstrated the extensive masking of crRNA (crRNA13a in Supplementary Table 1). We next examined the performance of chemical masking on the cleavage of target RNA (target13a in Supplementary Table 1). In this study, target RNAs were labeled at their 5′-ends with FAM to facilitate analysis. We observed significant masking-dependent inhibition of Cas13a cleavage (Fig. 3a). On the basis of these results, there was a direct interaction between the masking extent and CRISPR/Cas13a function.

Next, we examined whether Staudinger reduction can trigger the removal of masking group and thus restore the CRISPR/Cas13a function. To this purpose, increasing amounts of DPBM were added to CRISPR/Cas13a systems with masked crRNA13a and incubated further. Our strategic direction was fully supported. The removal of AMN groups by DPBM were both concentration-dependent and time-dependent (Supplementary Fig. 16B, C). The masking-induced blockage of RNA cleavage was gradually reduced in response to increasing concentrations of DPBM (Fig. 3b). Not surprsingly, the treatment with a high concentration of DPBM did not affect the cleavage with original crRNA13a (lane 3 in Fig. 3b).

Unlike DNA-targeting Cas9 enzymes, the Cas13a can remain active after cutting its crRNA-targeted single-stranded RNA and cleave non-targeted collateral RNA^10,40^. This feature allowed us to use fluorescence kinetic assay to further demonstrate our strategy^40^. A similar phenomenon, negative regulation of Cas13a cleavage by chemical masking, was observed (Fig. 3c, d). Evidently, chemical masking (200 mM NAI-N_3_, 2.0 h) almost completely aborted Cas13a functions. We further confirmed that the DPBM treatment reactivated collateral cleavage of Cas13a. The following results demonstrated a gradual recovery of RNA cleavage in response to increasing amounts of DPBM (Fig. 3e, f). Additionally, there appeared to be a slight decrease in the fluorescence intensity for the non-masked control treated with DPBM. One reason possibly was that phosphine compounds with an aromatic ring might interfere with the FAM fluorescence intensity by fluorescence quenching. These observations suggested that our strategy was efficient to regulate the CRISPR/Cas13a functions.

### RNA stability assay

**Fig. 4:**
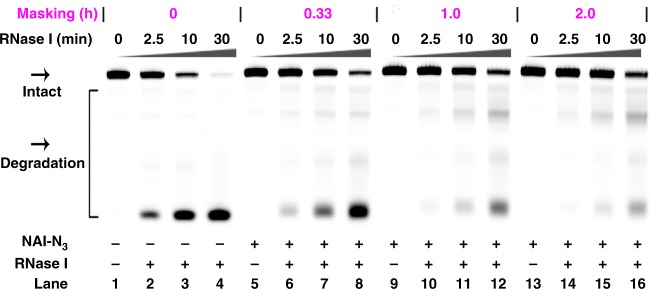
Chemical masking protects gRNA from RNase degradation. Reactions were carried out as described in the Experimental section. All samples were tested in three biological replicates. Lanes 1, 5, 9, 13: no RNase I control; lanes 2, 6, 10, 14: RNase I cleavage for 2.5 min; lanes 3, 7, 11, 15: RNase I cleavage for 10 min; lanes 4, 8, 12, 16: RNase I cleavage for 30 min. Source data is available in the Source Data file.

RNases are ubiquitous and even trace amounts of them are able to degrade a significant amount of RNA. However, very few studies emphasize CRISPR stability because in this system, gRNA easily undergo RNase cleavage^41^, especially when applied in vivo. We next addressed whether chemical masking could have effects in protecting gRNA from RNase cleavage. In this study, the different treatment gRNAs were subject to degradation by different RNases (RNase I and RNase T1). The representative data were shown here (Fig. 4 and Supplementary Fig. 17). For the unmodified crRNA13a, we observed a large portion of degraded products after RNase I degradation for 30 min (lane 4 in Fig. 4). Chemical masking (200 mM NAI-N_3_, 2.0 h) nearly completely blocked the cleavage of crRNA13a with RNase I (lanes 14–16 in Fig. 4). These observations suggest a potentially important role for chemical masking in promoting CRISPR stability, which is critical for future in vivo applications.

### Interactions of gRNA with both Cas13a and target RNA

**Fig. 5:**
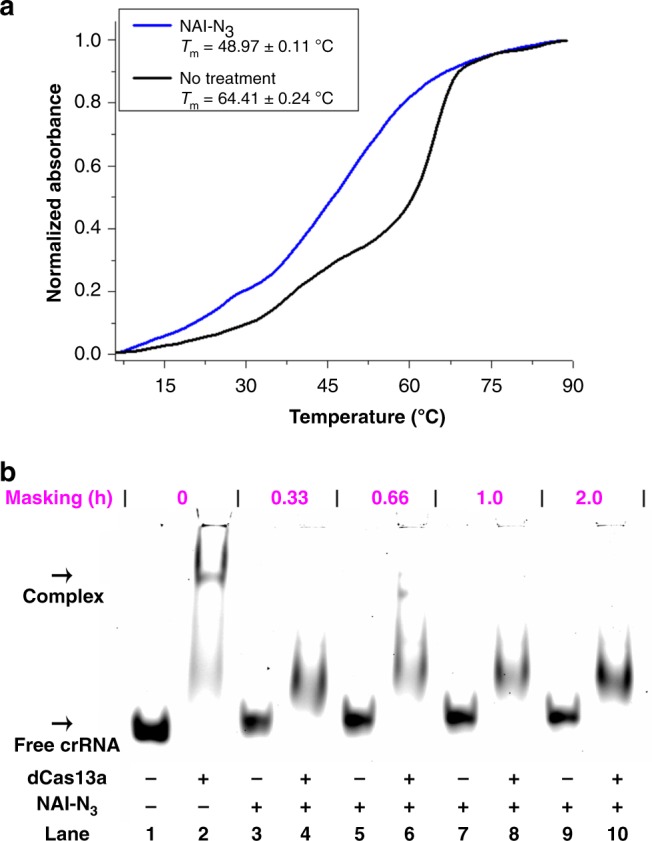
Interactions of crRNA13a with both Cas13a and target RNA. **a** Representative melting profiles of the crRNA/target RNA with different treatments were recorded in 10 mM Tris-HCl buffer (pH 7.0, 100 mM NaCl). Melting temperature of the NAI-N_3_-treated sample was statistically significantly different from that of the no treatment control (*P* < 0.05). **b** The dCas13a was incubated with 5′-Cy3-labeled crRNA probe (crRNA13a-Cy3 in Supplementary Table 1) with different treatments, and the samples were separated on native 6% PAGE gels. Lanes 1, 3, 5, 7, 9: no protein control; lanes 2, 4, 6, 8, 10: crRNA13a was incubated with a 5.0 molar excess of protein. Source data is available in the Source Data file.

We next explored potential mechanisms by which chemical masking functions to halt the CRISPR functions. The CRISPR/Cas13a system was examined for proof of concept study^10,36^. In common ideas, the interactions between crRNA/Cas13a and target RNA dominate the functions of CRISPR/Cas13a^36^. The UV melting study was performed to gain insights into intermolecular interactions between crRNA and target RNA. In this investigation, ultraviolet (UV) melting curves were recorded at 260 nm to monitor the dissociation of RNA duplexes. Figure 5a showed normalized UV melting curves of RNA duplexes with and without masking. Not surprisingly, chemical masking significantly decreased the stability of crRNA/target RNA duplex (Fig. 5a).

Next, we explored the effects of chemical masking on interactions between crRNA and Cas13a protein. In molecular biology, an electrophoretic mobility shift assay (EMSA) is a common technique to study protein-nucleic acid interactions. In this study, we used the LbuCas13a R1079A/K1080A double mutant (dCas13a)^10^. This mutant enzyme retained crRNA-binding ability, but was unable to cleave RNA targets. In this study, crRNAs were labeled at their 5′-ends with Cy3 to facilitate analysis. Prior to masking, the band corresponding to the crRNA/protein binary complex could be observed (lane 2 in Fig. 5b), whereas chemical masking significantly affects the recognition and binding of dCas13a protein to crRNA (lanes 4, 6, 8, and 10 in Fig. 5b).

We further examined the effects of chemical masking on interactions between gRNA and Cas9 protein. This study was performed by using fluorescently labeled nuclease-deficient Cas9 (dCas9), which was expressed and purified according to a previous literature^42^. Being consistent with the above results, chemical masking significantly blocked the ability of gRNA to bind dCas9 (Supplementary Fig. 18). Taken together, it was probable that the decreased binding affinity between gRNA and Cas protein and the decreased base pairing between gRNA and target sequence both contribute to the blockage of CRISPR functions.

### Conditional control of gene editing in live cells

Having demonstrated the effectiveness of our strategy in controlling nucleic acid cleavage, we became interested in exploring its application in live cells. As a proof-of-concept demonstration we chose to study the endogenous *HBEGF* (heparin binding EGF like growth factor) gene and *ANTXR1* (Anthrax toxin receptor 1) in Cas9 expressing stable HeLa cells (HeLa-OC cells)^43^. We selected these genes which have been targeted with individual gRNAs (5′-UTR targeting gRNA-HBEGF and gRNA-ANTXR1 in Supplementary Table 1) with high editing frequencies in cells (schematic illustration in Supplementary Figs. 19, 20)^43^. Our in vitro DNA cleavage assay demonstrated that the cleavage of target HBEGF and ANTXR1 DNA can be regulated using our strategy (Supplementary Figs. 21–24). Additionally, we examined the cleavage of two off-target sites known to be targeted by the gRNA-HBEGF RNA (Schematic illustration in Supplementary Fig. 25). Interestingly, our in vitro study demonstrated that chemically unmasked CRISPR systems retained a similar efficiency to cut the on‐target sequence but with an evidently decreased ability to cut the off‐target sequence (Supplementary Fig. 26). Therefore, the intermediate gRNAs (partially unblocked) exhibit compromised off-target DNA cleavage. We next delivered individual gRNAs into HeLa-OC cells by lipofectamine 3000. Followingly, a T7E1 nuclease assay was performed to measure the percentage of indel (insertion/deletion mutation) according to previous protocols^43^. The results indicated that the delivery of original gRNAs could induce targeted indels indicative of mutagenic non-homologous end joining (NHEJ) in HeLa-OC cells (lane 3 in Supplementary Fig. 27A, B). We further demonstrated that in-cell CRISPR functions can be inhibited by chemical masking of gRNA (lanes 4–6 in Supplementary Fig. 27A, B).

**Fig. 6:**
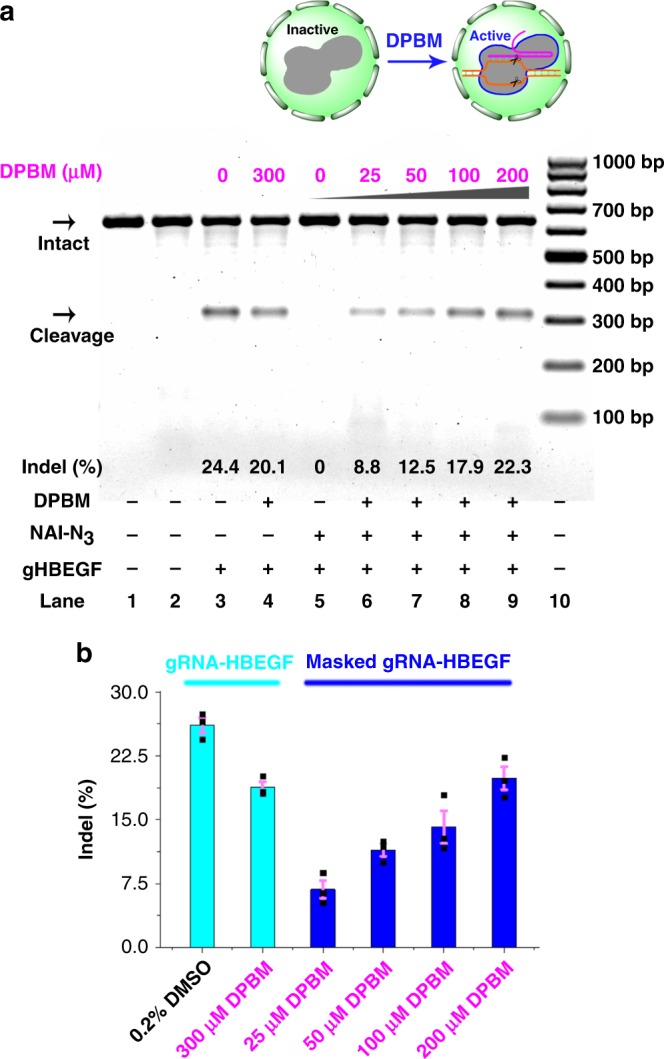
Gene editing in live cells. The T7E1 nuclease assay was performed 24 h post-transfection using Cas9-expressing HeLa-OC cells transfected with gRNA-HBEGF with different treatments. **a** Uncleaved HBEGF DNA (621 bp) cut to shorter cleavage fragments (311 bp and 310 bp) were indicated. Lane 1: target control; lane 2: no gRNA-HBEGF control; lane 3: original gRNA-HBEGF; lane 4: original gRNA-HBEGF, 300 μM DPBM; lanes 5–9: masked gRNA-HBEGF (200 mM, 1 h), different concentrations of DPBM; lane 10: DNA markers. The “Lanes and Bands” tool in Image Lab software version 5.1 (Bio-Rad) was used for image acquisition and differential densitometric analysis of the associated bands from the gels. Although long exposure times were necessary to visualize faint bands, the calculations of indel formation were not influenced by the extent of exposure. **b** The Cyan color represents the original gRNA-HBEGF group, while the Blue color represents the masked gRNA-HBEGF group. Error bars: ±SEM. Source data are provided as a Source Data file.

The next important issue we studied was whether gene editing can be reactivated with in cell Staudinger reduction. Cell proliferation assays demonstrated that DPBM treatments did not significantly reduce the viability of cultured HeLa-OC cells (Supplementary Fig. 28). Individually masked gRNAs were then delivered into cells as above before increasing amounts of DPBM were added. Indeed, we observed that targeted indel frequencies increased in a dose-dependent manner (Fig. 6 and Supplementary Fig. 29). We wished to characterize the kinetics of in cell Staudinger reaction. Of interest, the unblocking reagent can be removed from cells after 2 h of incubation (Supplementary Fig. 30). Moreover, the kinetics were not affected by the sequence of the gRNAs (Supplementary Fig. 31). Since the the current method was based on temporal control, we performed an experimental comparison to another one with temporal control (CRISPR-plus)^30^. These two methods were substantially different. Although both methods worked well for manipulating cell-free DNA in test tubes (Supplementary Figs. 32, 33), our results provided evidence that the current method produced a better off/on switch of gene editing in cells (Supplementary Fig. 34). From these observations, in-cell CRISPR functions can be regulated effectively through chemical activation of gRNAs.

## Discussion

After discovery, CRISPR systems have extensive applications in DNA and RNA technology^21,44–46^. The issue of constitutive function and off-target effects poses the main obstacles and challenges for further application of sequence-based CRISPR tools^18,47,48^. We therefore aimed at obtaining spatio control of CRISPR systems. However, this is not an easy task, requiring additional genes and inducers^18,19,21,49^, additional transacting factors^50^, or even invasive approaches to limit the exposure time of the genome to active CRISPR system (Supplementary Table 2)^51^. Related studies have been largely concentrated on modifying the Cas enzyme, such as light-activated or chemical-activated reassembly of a split Cas enzyme^25,52,53^, site-specific modification of Cas proteins^24,26^, or intein-mediated protein splicing^20,54^. In regard to “simple is beautiful,” we developed an easy and reliable model for the transient chemical control of CRISPR systems. Chemical manipulation of guide RNA confers to the researcher an advantageous alternative for our purpose. In theory, the current method is applicable to virtually any given guide RNA of interest.

The present study indicates that this strategy enabled us to control nucleic acid cleavage and even provide temporal control over gene editing in human cells. In our demonstrated examples, the CRISPR functions were tightly controlled in response to conditions both inside and outside cells. Importantly, the CRISPR systems with masked gRNA were completely inactive, and its activity can be restored up to native levels through a brief Staudinger reduction. These results together suggested that chemical intervention of CRISPR functions was a consequence of the disruption of contacts between gRNA/Cas and target nucleic acids. Previous studies have demonstrated the roles of a preformed ribonucleoprotein (RNP) complex in improving RNA stability and cellular entry^55^. Delivery of purified Cas9 protein and gRNA as an RNP complex has recently emerged as a useful approach to genome editing^56–58^. Since dCas9 cannot bind masked RNA, then this eliminates the possibility of RNP delivery of Cas9/masked gRNA into cells. Hence, our strategy was not compatible with CRISPR RNP preparation. Importantly, our method offers a complementary approach to the current strategy to regenerate the activity of Cas protein with a specific masked amino acid residue on its backbone^24^. Although our strategy is yet to be tested in animal models, the robustness of this strategy and the biocompatibility of Staudinger reduction underline its therapeutic potential.

In summary, we have developed a method to regulate nucleic acid cleavage and gene editing in live cells. The simplicity and efficiency of this strategy may enable its rapid development as a tool to versatile applications in chemical biology.

## Methods

### Materials

Cas9 Nuclease, *Streptococcus pyogenes* (product# M0646), T7 Endonuclease I (product# M0302) Ribonucleotide solution mix (NTPs) and deoxy-ribonucleoside triphosphates (dNTPs) were purchased from New England Biolabs (USA). Transcript Aid T7 High Yield Transcription kit (product# K0441) and Glycogen (product# R0561) were purchased from Thermo Fisher Scientific. Pyrobest™ DNA Polymerase and PrimeSTAR HS DNA Polymerase were purchased from TaKaRa Shuzo Co. Ltd. (Tokyo, Japan). DNA Clean & Concentrator™-5 kit (product# D4014) was purchased from Zymo Research Corp. The DNeasy Blood & Tissue Kit was purchased from QIAGEN. The oligonucleotides at HPLC purity were obtained from TaKaRa company (Dalian, China). The nucleic acid stains Super GelRed (NO.: S-2001) was bought from US Everbright Inc. (Suzhou, China). DPBA (CAS# 17261-28-8), TCEP (CAS# 51805-45-9), 4-(2-Hydroxyethyl)piperazine-1-ethanesulfonic acid (HEPES, CAS# 7365-45-9) and Thiazolyl Blue Tetrazolium Bromide (MTT, CAS# 298-93-1) were purchased from Sigma-Aldrich Inc. (Shanghai, China). DPBS (CAS# 63995-75-5) was purchased from TCI Development Co., Ltd (Shanghai). The pH was measured with Mettler Toledo, FE20-Five Easy™ pH (Mettler Toledo, Switzerland). The concentration of DNA or RNA was quantified by NanoDrop 2000c (Thermo Fisher Scientific, USA). Gel Imaging was performed using Pharos FX Molecular imager (Bio-Rad, USA).

### Chemical synthesis

The synthesis of NAI-N_3_, mNAI, and DPBM was performed according to previous literatures^59,60^. Since NAI-N_3_ and phosphines were easily oxidized on standing for long periods of time, the stock solutions were prepared fresh just prior to each use.

### In vitro transcription and purification of gRNA

The gRNA to different targets were synthesized by in vitro transcription with T7 RNA polymerase according to manufacturer’s protocol. To generate the template for gRNA, we designed separate shorter overlapping sequences^30^. In each forward primer, a T7 promoter sequence was flanked on the 5′-end of target region of gRNA (Supplementary Table 1). A brief template-free PCR was performed to anneal and fill in these overlapping sequences using Pyrobest™ DNA Polymerase. The full-length dsDNA template was then PCR amplified to generate the gRNA encoding DNA template. Transcription reactions were performed at 37 °C for 4.0 h in 1× Transcript aid reaction buffer containing 100 mM HEPES-KOH (pH 7.9), 20 mM MgCl_2_, 30 mM DTT, 2 mM each NTP, 2 mM spermidine, 0.1 mg/mL T7 RNA polymerase, and 300 ng PCR fragments. Following DNA degradation using DNase I at the end of the transcription, the transcribed RNA products were then purified using the NaOAc/phenol/chloroform method. The purified gRNA was resuspended in RNase-free H_2_O.

### Post-synthetic masking of gRNA

This assay was performed in 1× masking buffer, which contained 100 mM HEPES, 6 mM MgCl_2_, 100 mM NaCl at pH 7.5@ 25 °C^34^. Prior to masking, gRNA (20 μM) was incubated at 95 °C for 2 min and fast cooled to 4 °C in 1× masking buffer. The masking was started by adding 1.0 μL NAI-N_3_ solution in DMSO (2.0 M) to a total volume of 10 μL mixture. After incubated at 37 °C for various durations, 1.0 μL NaOAc (3 M), 37.5 μL ethanol and 1.0 μL glycogen (5 mg/mL) was added into the mixture to quench the reaction. The sample was placed at −80 °C for 3.0 h and centrifuged at 12,000 × *g* for 1.0 h subsequently. The resulting RNA pellets were rinsed twice with 75% ethanol, then dried and resuspended in RNase-free water.

### Unmasking of acylated gRNA

Acylated gRNA (1 μM) was incubated in 50 mM Tris-HCl buffer (pH 7.5) containing 10% DMSO with each phosphine at different concentrations at 37 °C. The reaction was stopped by addition of 10% v-v of NaOAc (3 M) followed by 3.75× v:v of ethanol and 1.0 μL glycogen (5 mg/mL). The following procedure for product purification was similar to the above one.

### Conditional control of RNA-guided DNA cleavage

Target GFP or HBEGF fragments were PCR amplified from pEGFP-C1 vector (Clontech) or genomic DNA (HeLa-OC cells) using the following PCR primers: t-GFP-1F and t-GFP-1R for t-GFP1; t-GFP-2F and t-GFP-2R for t-GFP2; t-HBEGF-F and t-HBEGF-R for t-HBEGF; t-ANTXR1-F and t-ANTXR1-R for t-ANTXR1; t-OT1-HBEGF-F and t-OT1-HBEGF-R for t-OT1-HBEGF; t-OT2-HBEGF-F and t-OT2-HBEGF-R for t-OT2-HBEGF. In vitro Cas9 cleavage assay was performed in 1× NEBuffer™ 3.1, which contained 50 mM Tris-HCl, 100 mM NaCl, 10 mM MgCl_2_ and 100 μg/mL BSA at pH 7.9 @ 25 °C. Briefly, 50 ng of gRNA, 100 ng of target DNA, 1.0 μg of BSA and Cas9 (1.0 U) were incubated with 10 μL of 1× NEBuffer™ 3.1 buffer in RNase free water at 37 °C according to the manufacturer’s protocol.

For CRISPR blockage assay, the gRNAs (50 ng) with different acylation degrees were mixed with target DNA fragments (100 ng), and subjected to an enzymatic digest with Cas9 at 37 °C for 24 h. Reactions were quenched by adding SDS containing loading dye and loaded onto a 1.5% agarose gel containing 1.5× Super GelRed for visualization (100 V, 1.5 h). Original gRNA without acylation was used in control reactions.

For CRISPR activation assay, the gRNA (50 ng) with the desired masking level was mixed with Cas9. The reaction was started by rapid mixing of equal volumes of the Cas9:gRNA complex with an solution containing target DNA fragments (100 ng) and two fold concentrations of phosphine in 1× NEBuffer™ 3.1 buffer. The reaction mixture was incubated at 37 °C for 24 h (unless otherwise indicated). The following procedure is similar to the above one.

### LbuCas13a expression and purification

The wildtype and the mutant *LbuCas13a* gene (Plasmid #83482, #91861) were bought from Addgene^10^. Each fusion protein contained an N-terminal His6-MBP-TEV protease cleavage site. Each protein was overexpressed in *E. coli* BL21 (DE3) (Novagen) cells that were induced with 0.2 mM isopropyl-1-thio-b-d-galactopyranoside (IPTG) at OD600 = 0.6 for 14 h at 16 °C. Cells were collected and lysed by sonication in a buffer containing 25 mM Tris-HCl, pH 8.0, 150 mM NaCl. Upon centrifugation, the supernatant was incubated with Ni Sepharose (GE Healthcare), and the bound protein was eluted with a buffer containing 25 mM Tris-HCl, pH 8.0, 150 mM NaCl and 250 mM imidazole. Each eluted protein was digested with TEV protease at room temperature for 3 h to remove the His6-MBP tag, and then further purified using ion exchange chromatography on a prepacked HiTrap Heparin HP column (GE Healthcare), eluting with a buffer containing 25 mM Tris-HCl, pH 8.0, 1 M NaCl. Each protein was further purified by size-exclusion chromatography (Superdex 200 Increase 10/300, GE Healthcare) in a buffer containing 25 mM Tris-HCl, pH 8.0, 150 mM NaCl, and then concentrated to a concentration of 10 mg/mL.

### Conditional control of RNA-guided RNA cleavage

In vitro Cas13a cleavage assay was performed in 1× Cas13a buffer in RNase free water, which contained 20 mM HEPES, 50 mM KCl, 5 mM MgCl_2_ and 5% glycerol at pH 6.8 @ 25 °C. The crRNA guides were pre-folded by heating to 65 °C for 5 min and then slowly cooling to room temperature in 1× Cas13a buffer. Briefly, crRNA13a was complexed with a 2.0 molar excess of protein in 1× Cas13a buffer at 37 °C for 10 min, before adding 5′-FAM labeled RNA targets.

For CRISPR blockage assay, the crRNA13a with different degrees of acylation was mixed with RNA targets, and subjected to an enzymatic digest with Cas13a. Final concentration of all components was as following: 45 nM purified Cas13a protein, 22.5 nM crRNA13a, 150 nM 5′-end labeled target RNA. Reactions were allowed to proceed for 2 h at 37 °C (unless otherwise indicated). Reactions were stopped by addition of a 4.5-fold excess of quenching solution (95% formamide, 25 mM EDTA at pH 8.0), and samples were analyzed on a denaturing 20% polyacrylamide gel (400 V, 30 min). After electrophoresis, the oligonucleotides in the gel were visualized using a Pharos FX Molecular imager (Bio-Rad, USA) in the fluorescence mode (λex = 488 nm). The original crRNA13a without any treatment is used as the control.

For CRISPR activation assay, the crRNA13a (45 nM) with the desired acylation level was mixed with purified Cas13a (90 nM). The reaction was started by rapid mixing of equal volumes of the Cas13a:crRNA complex with an solution containing RNA targets (300 nM) and two fold concentrations of DPBM in 1× Cas13a buffer. The reaction mixture was incubated at 37 °C for 2 h. The following procedure is similar to the above one.

### The Cas13a collateral-cleavage assay

This assay was performed with 45 nM purified LbuCas13a, 22.5 nM crRNA13a, 22.5 nM target RNA, 125 nM quenched fluorescent RNA reporter (reporter1 in Supplementary Table 1) and 0.5 U RiboLock RNase inhibitor (Thermo Fisher Scientific), in 1× Cas13a buffer^10^. The curve describing fluorescence versus time was determined using an F-4600 FL Spectrophotometer (Hitachi) equipped with a xenon lamp with the kinetics mode at room temperature. For kinetic measurement, RNA reporter at a final concentration of 125 nM was added at time (*t*) = 0. Reactions were allowed to proceed for 1 hr measured every 1 s. The excitation and emission wavelengths are set to 496 and 520 nm and a 1-cm path-length cell is used. Slit width: excitation = 10 nm; emission = 10 nm. The gRNA without any treatment is used as an internal control.

### gRNA stability assay (RNase I)

This assay was performed in 1× buffer in PCR grade water, which contained 20 mM Tris-acetate, 100 mM NaCl, 0.1 mM EDTA and 0.01% Triton X-100 at pH 8.0 @ 25 °C. The crRNA13a-Cy3 (100 ng) with or without masking was incubated in the presence of 0.01 U RNase I in a reaction volume of 10 μL at 37 °C for various durations. Reaction was quenched by adding a 4.0-fold excess of quenching solution (0.1% SDS in formamide). RNA products were immediately separated on a denaturing 20% polyacrylamide gel (350 V, 1.0 h).

### UV melting studies

The UV melting experiments were carried out using a Jasco-810 spectropolarimeter (Jasco, Easton, MD, USA) equipped with a water bath temperature-control accessory. The detection is performed with a quartz cell (optical path length at 1 mm). crRNA/target RNA duplex (5.0 μM) was incubated in 10 mM Tris-HCl buffer (pH 7.0) containing 50 mM NaCl. The UV melting profiles were recorded by using a heating rate of 0.2 °C/min and the absorbance values were recorded every 1 °C at a wavelength of 260 nm. The melting point (Tm) corresponds to the midtransition temperature, which was determined by the maximum of the first derivative of the absorbance as a function of temperature.

### EMSA assay

Assays were carried out in 1× EMSA buffer, which contained 20 mM HEPES, 50 mM KCl, 10 μg/mL BSA, 100 μg/mL yeast tRNA, 0.01% Igepal CA-630 and 5% glycerol at pH 6.8 @ 25 °C^10^. crRNA13a-Cy3 with or without masking was incubated with the dCas13a for 10 min at 37 °C. Samples were then resolved by 6% native PAGE at 4 °C (0.5× TBE buffer). After electrophoresis (100 V, 1.0 h), in gel targets were visualized using a Pharos FX Molecular imager (Bio-Rad, USA) in the fluorescence mode (λex = 590 nm).

### Gene editing in live cells

Human HeLa-OC cell line was a kind gift from Prof. Wen-Sheng Wei, Biodynamic Optical Imaging Center (BIOPIC), School of Life Sciences, Peking University, Beijing, China. HeLa-OC cells were cultured in complete media, Gibco™ DMEM (Dulbecco’s modification of Eagle medium), High Glucose medium (Thermo Fisher Scientific), 10% (v/v) Gibco™ fetal bovine serum (FBS, Thermo Fisher Scientific) and 1% penicillin/streptomycin (Invitrogen), at 37 °C in a 5% CO_2_ incubator^43^. Cells (4 × 10^5^ per well) were seeded into 6-well plates overnight before transfection and washed twice with DPBS (Dulbecco’s Phosphate-Buffered Saline), and 300 μL of pre-warmed DMEM was added to each well. Different gRNAs (2.5 μg at a concentration of 0.5 μg/μL) were mixed in 120 μL of DMEM. The Lipofectamine 3000 transfection agent (5.0 μL, Thermo Fisher Scientific) in 120 μL of DMEM per well were added to the diluted gRNA, followed by an incubation (10 min). The complex was added to the cells, and the medium was changed to complete DMEM after an incubation (6 h) at 37 °C in 5% CO_2_. Cells were further cultured for different periods at 37 °C. Genomic DNAs were extracted for mutation detection using Qiagen DNeasy Blood and Tissue Kit according to manufacturer’s protocol. Subsequently, target fragments containing target sites were amplified from genomic DNA (200 ng) using PrimeSTAR HS DNA Polymerase with primer sets (tHBEGF-F, tHBEGF-R, t-ANTXR1-F, t-ANTXR1-R in Supplementary Table 1). The cycling conditions are as following: initial denaturation at 94 °C for 15 s; 35 cycles consisting of 10 s of denaturation at 98 °C, 1 min of annealing and extension at 68 °C. The amplified DNA products were purified via Zymo Research DNA Clean and Concentrator Kit according to manufacturer’s protocol. T7 Endonuclease I digestion of DNA substrates carrying the target loci (100 ng) was performed according to manufacturer’s protocol. Reaction was quenched by adding SDS containing loading dye and loaded onto a 1.5% agarose gel containing 1.5× Super GelRed for visualization (100 V, 1.5 h).

For CRISPR blockage assay, cells were transfected with gRNA with different masking levels. The following procedure is similar to the above one.

For CRISPR activation assay, cells were transfected with masked gRNA (200 mM NAI-N_3_, 1 h). The following procedure was similar to the above one wherein DPBM was added sequentially to cell culture right after replacing transfection complexes.

### Statistical analysis

Statistical analysis was performed using ORIGIN 8.5 software. The differences were considered to be significant for *P* < 0.05.

### Reporting summary

Further information on research design is available in the Nature Research Reporting Summary linked to this article.

## Supplementary information

Supplementary Information

Reporting Summary

## Data Availability

All relevant data supporting the key findings of this study are available within the article and its Supplementary Information files or from the corresponding author upon reasonable request. Source data is available in the Source Data file.

## Source data

Source Data
