# Supplementary material for: Conditional control of RNA-guided nucleic acid cleavage and gene editing
Source: Nat Commun. 2020 Jan 3;11:91. doi: 10.1038/s41467-019-13765-3 (PMC6941951; doi:10.1038/s41467-019-13765-3)
Supplement: Supplementary file 1 — Supplementary Information [file 41467_2019_13765_MOESM1_ESM.pdf]

Supporting Information

Conditional control of RNA-guided nucleic acid cleavage and gene editing

Wang, S.R. et al.

Supplementary Notes

Materials

Cas9 Nuclease, *Streptococcus pyogenes* (product# M0646), T7 Endonuclease I (product# M0302) Ribonucleotide solution mix (NTPs) and deoxy-ribonucleoside triphosphates (dNTPs) were purchased from New England Biolabs (USA). Transcript Aid T7 High Yield Transcription kit (product# K0441) and Glycogen (product# R0561) were purchased from Thermo Fisher Scientific. Pyrobest™ DNA Polymerase and PrimeSTAR HS DNA Polymerase were purchased from TaKaRa Shuzo Co. Ltd. (Tokyo, Japan). DNA Clean & Concentrator™-5 kit (product# D4014) was purchased from Zymo Research Corp. The DNeasy Blood & Tissue Kit was purchased from QIAGEN. The oligonucleotides at HPLC purity were obtained from TaKaRa company (Dalian, China). The nucleic acid stains Super GelRed (NO.: S-2001) was bought from US Everbright Inc. (Suzhou, China). DPBA (CAS# 17261-28-8), TCEP (CAS# 51805-45-9), 4-(2-Hydroxyethyl)piperazine-1-ethanesulfonic acid (HEPES, CAS# 7365-45-9) and Thiazolyl Blue Tetrazolium Bromide (MTT, CAS# 298-93-1) were purchased from Sigma-Aldrich Inc. (Shanghai, China). DPBS (CAS# 63995-75-5) was purchased from TCI (Shanghai) Development Co., Ltd. The pH was measured with Mettler Toledo, FE20-Five Easy™ pH (Mettler Toledo, Switzerland). The concentration of DNA or RNA was quantified by NanoDrop 2000c (Thermo Scientific, USA). Gel Imaging was performed using Pharos FX Molecular imager (Bio-Rad, USA).

Chemical synthesis

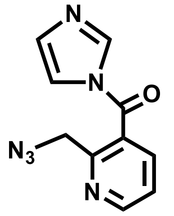

2-azidomethylnicotinic acid imidazolide
(NAI-N_3)

The NAI-N_3 was synthesized according to a previous literature[1].

^{1}H NMR (400 MHz, DMSO-d_6) δ 8.85 (dd, *J* = 4.9, 1.7 Hz, 1H), 8.16 (m, 2H), 7.67 (m, 1H), 7.61 (dd, *J* = 7.8, 4.9 Hz, 1H), 7.18 (dd, *J* = 1.7, 0.8 Hz, 1H), 4.63 (s, 2H).

^{13}C NMR (101 MHz, DMSO-d_6) δ 165.24, 155.12, 152.38, 139.00, 138.19, 131.38, 127.77, 123.46, 118.36, 53.22.

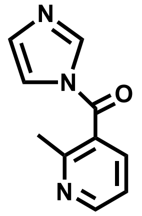

2-methylnicotinic acid imidazolide

(mNAI)

The mNAI was synthesized according to a previous literature[1].

^{1}H NMR (400 MHz, $CDCl_3$) δ 8.75 (dd, *J* = 4.9, 1.7 Hz, 1H), 7.91 (s, 1H), 7.76 (dd, *J* = 7.8, 1.7 Hz, 1H), 7.45 (t, *J* = 1.5 Hz, 1H), 7.33 (dd, *J* = 7.7, 5.1 Hz, 1H), 7.18 (dd, *J* = 1.5, 0.6 Hz, 1H), 2.63 (s, 3H).

^{13}C NMR (101 MHz, $CDCl_3$) δ 165.28, 157.16, 152.10, 137.65, 135.93, 131.68, 127.77, 120.63, 117.18, 23.00.

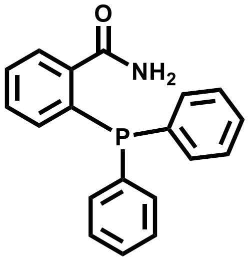

2-(diphenylphosphanyl)benzamide

(DPBM)

The DPBM was synthesized according to the previous literature[2].

^{1}H NMR (400 MHz, $CDCl_3$) δ 7.67 (ddd, *J* = 7.5, 3.8, 1.5 Hz, 1H), 7.43 – 7.23 (m, 12H), 6.98 (ddd, *J* = 7.6, 4.0, 1.4 Hz, 1H), 6.07 (s, 1H), 5.79 (s, 1H).

^{13}C NMR (101 MHz, $CDCl_3$) δ 170.65, 139.97, 139.73, 137.01, 136.91, 136.71, 136.49, 134.30, 134.00, 133.80, 130.64, 128.89, 128.74, 128.66, 128.59, 128.11, 128.06.

Denaturing polyacrylamide gel electrophoresis

The denaturing polyacrylamide gel electrophoresis was run in a temperature-controlled vertical electrophoretic apparatus (DYCZ-22A, Liuyi Instrument Factory, Beijing, China). The acrylamide concentration of the separating gel was 20 % (19 : 1 monomer to bis ratio). About one hundred nanograms of DNA were loaded on the gel. Electrophoresis was run at 10 °C for 12 hr at 400 V. The Super GelRed was used for post-electrophoresis gel staining. The target nucleic acids in the gel were visualized using a Pharos FX Molecular imager (Bio-Rad, USA) in the fluorescence mode (λex = 590 nm).

gRNA stability assay (RNase T1)

This assay was performed in 1 × buffer in PCR grade water, which contained 50 mM Tris-HCl and 2 mM EDTA at pH 7.5 @ 25 °C. The 5'-Cy3 labeled crRNA13a (crRNA13a-Cy3, 100 ng)

with or without masking was incubated in the presence of 1.5 U RNase T1 in a reaction volume of 10 μL at 37 °C for various durations. Reaction was quenched by adding a 4.0-fold excess of quenching solution (0.1% SDS in formamide). RNA products were immediately separated on a denaturing 20% polyacrylamide gel (350 V, 1.0 hr).

dCas9 expression and purification[3]

The *pET302-6His-dCas9-Halo* gene (Plasmid #72269) was bought from Addgene. dCas9 protein was overexpressed in *E. coli* BL21 (DE3) (Novagen) cells that were induced with 0.5 mM isopropyl-1-thio-b-D-galactopyranoside (IPTG) at OD600 = 0.6 for 15 hr at 18 °C. Cells were collected and lysed by sonication in a lysis buffer containing 50 mM Na_3PO_4, pH 7.0, 300 mM NaCl. Upon centrifugation, the supernatant was incubated with Ni Sepharose (GE Healthcare), and the bound protein was eluted with an elution buffer containing 50 mM Na_3PO_4, pH 7.0, 300 mM NaCl and 150 mM imidazole. The eluted protein was exchanged into buffer [50 mM HEPES (pH 7.5), 100 mM KCl, 1 mM DTT] by a 50-K molecular weight cut off (MWCO) centrifugal filter (Millipore Amicon). The protein was further purified on the ÄKTA FPLC system by ion exchange chromatography using a prepacked HiTrap SP HP column (GE Healthcare), eluting with a linear gradient of buffers containing 0-1.0 M KCl. The protein was further purified by size-exclusion chromatography (Superdex 200 Increase 10/300, GE Healthcare) in a buffer containing 50 mM HEPES, pH 7.5, 150 mM KCl, and then concentrated to a concentration of 10 mg per mL.

EMSA assay (dCas9)[3]

For Halo domain labeling, fluorescent Halo ligand (G1002 HaloTag® Alexa Fluor® 488 ligand, Promega) was incubated with dCas9 at ratio of 10:1 at room temperature for 30 min followed by incubation at 4 °C overnight. The excessive unreacted fluorescent Halo ligand was removed using 40-K MWCO Zeba spin desalting columns (Thermo Scientific). Assays were carried out in 1 × EMSA buffer, which contained 20 mM HEPES, 50 mM KCl, 10 μg per mL BSA, 100 μg per mL yeast tRNA, 0.01% Igepal CA-630 and 5% glycerol at pH 6.8 @ 25 °C[3]. dCas9 protein and gRNA (with or without masking) were incubated together at room temperature for 15 min to examine the binary complex. Samples were then resolved by 6% native PAGE at 4 °C (0.5 × TBE buffer). After electrophoresis (100 V, 1.0 hr), in gel targets were visualized using a Pharos FX Molecular imager (Bio-Rad, USA) in the fluorescence mode (λex = 488 nm).

Cell survival assay

Human HeLa-OC cells were grown in Gibco™ DMEM (Dulbecco’s modification of Eagle medium), High Glucose medium (Thermo Fisher Scientific) containing 10% (v/v) Gibco™ fetal bovine serum (FBS, Thermo Fisher Scientific) and 1% penicillin/streptomycin (Invitrogen) at 37 °C, 5% CO_2 humidified atmosphere. HeLa-OC cells (5×10^4) were seeded into wells of a 96-well plate and incubated for 16 hr. Then cells were treated with different amounts of freshly dissolved DPBM or DMSO control for 24 hr. After centrifugation at 2000 rpm for 10 min, the medium was discarded and fresh medium was added. Subsequently, MTT (20 μL, 5 mg per mL in

DMSO) was added to each well and the mixture was incubated for an additional 4 hr. After centrifugation at 2000 rpm for 10 min, the medium was removed. Subsequently, DMSO (150 μL) was added to each well and the OD values at 570 nm were detected by a microplate reader (SpectraMax M5, Molecular Devices) to evaluate cell viability.

The BrdU (5-Bromo-2-deoxyUridine) assay was performed according to the manufacturer's protocol (Abcam, ab126556). HeLa-OC cells (5×10^4) were seeded into wells of a 96-well plate and incubated for 16 hr. Then cells were treated with different amounts of freshly dissolved DPBM or DMSO control for 24 hr. BrdU (10 μL per well, 10 μM) was added and incubated for 4 hr at 37 °C. The cells were then fixed with fixing solution (3.7% formaldehyde in 1 × PBS, 1 mM KH_2PO_4, 155 mM NaCl, 3 mM Na_2HPO_4 @ pH 7.4) at room temperature for 30 min. After the fixing solution was removed, anti-BrdU monoclonal antibody conjugated with peroxidase (100 μL per well) was added and incubated at room temperature for 1 hr. Then, the cells were washed with 1 × PBS, and peroxidase-conjugated goat anti-mouse immunoglobulin G (IgG) antibody (1:2,000) was added and incubated for 30 min. Subsequently, the cells were washed with 1 × PBS and incubated with the peroxidase substrate solution for 30 min at room temperature in the dark. Finally, 100 μL of stop solution was added, and the absorbance was monitored at 450 nm by a microplate reader.

Light control of RNA-guided DNA cleavage[4]

The Protector DNA containing PhotoCleavable linkers (PC linkers) were obtained from TaKaRa company (Dalian, China). Individual gRNA was annealed with Protector complement at a molar ratio of 1:2 in annealing buffer, which contained 50 mM Tris-HCl, 100 mM NaCl and 10 mM $MgCl_2$ at pH 7.9 @ 25°C. Desired amount of gRNA/Protector in reaction mixture was irradiated for varied periods using a Spectroline UV source (UV-365EH model, 365 nm) at a distance of 4 cm from the source to test activity, followed by a further incubation at 37 °C for 24 hr (unless otherwise indicated). Reactions were quenched by adding SDS containing loading dye and loaded onto a 1.5% agarose gel containing 1.5 × Super GelRed for visualization (100 V, 1.5 hr).

Light control of gene editing

Human HeLa-OC cells (4×10^5 per well) were seeded into 6-well plates overnight before transfection and washed twice with DPBS, and 300 μL of pre-warmed DMEM was added to each well. Different gRNA/Protector complex (7.5 μg at a concentration of 1.5 μg per uL) were mixed in 120 μL of DMEM. The Lipofectamine 3000 transfection agent (5.0 μL, Thermo Fisher Scientific) in 120 μL of DMEM per well were added to the diluted gRNA, followed by an incubation (10 min). The complex was added to the cells, and the medium was changed to complete DMEM after an incubation (6 hr) at 37 °C in 5 % CO_2. Cells were irradiated using a Spectroline UV source (UV-365EH model, 365 nm) and further cultured for 24 hr at 37 °C. Genomic DNAs were extracted for mutation detection using Qiagen DNeasy Blood and Tissue Kit according to manufacturer's protocol. Subsequently, target fragments containing target sites were

amplified from genomic DNA (200 ng) using PrimeSTAR HS DNA Polymerase with primer sets (tHBEGF-F and tHBEGF-R in Supplementary Table 1). T7 Endonuclease I digestion of DNA substrates carrying the target loci (100 ng) was performed according to manufacturer's protocol. Reaction was quenched by adding SDS containing loading dye and loaded onto a 1.5% agarose gel containing 1.5 × Super GelRed for visualization (100 V, 1.5 hr).

<table>
<tr><th>Oligomer</th><th>Sequence(from 5’to 3’)</th><th colspan="2">Construct</th></tr>
<tr><td>gRNA-GFP-F</td><td>5’-TCTAATACGACTCACTATAGGGATGCCGTTCTTCTGCTTGTGTTTTAGAGCTAGAAATAGCA-3’</td><td rowspan="2">For gRNA construct (gRNA-GFP)</td><td></td></tr>
<tr><td>gRNA-R</td><td>5’-AAAAGCACCGACTCGGTGCCACTTTTTCAAGTTGATAACGGACTAGCCTTATTTTAACTTGCTATTTCTAGCTCTAAAAC-3’</td><td rowspan="2">For gRNA construct (gRNA-HBEGF)</td></tr>
<tr><td>gRNA-HBEGF-F</td><td>5’-TCTAATACGACTCACTATAGGGTTCTCTCGGCACTGGTGACGTTTTAGAGCTAGAAATAGCA-3’</td><td></td></tr>
<tr><td>gRNA-GFP+4-F</td><td>5’-TCTAATACGACTCACTATAGGGATGCATGCCGTTCTTCTGCTTGTGTTTTAGAGCTAGAAATAGCA-3’</td><td colspan="2">The forward primer for gRNA construct (gRNA-GFP+4)</td></tr>
<tr><td>gRNA-GFP+8-F</td><td>5’-TCTAATACGACTCACTATAGGGATGCATGCATGCCGTTCTTCTGCTTGTGTTTTAGAGCTAGAAATAGCA-3’</td><td colspan="2">The forward primer for gRNA construct (gRNA-GFP+8)</td></tr>
<tr><td>gRNA-ANTXR1-F</td><td>5’-TCTAATACGACTCACTATAGGGAGCGGAGAGCCCTCGGCATGTTTTAGAGCTAGAAATAGCA-3’</td><td colspan="2">The forward primer for gRNA construct (gRNA-ANTXR1)</td></tr>
<tr><td>gRNA-GFP</td><td>5’-GGGAUGCCGUUCUUCUGCUUGUGUUUUAGAGCUAGAAAUAGCAAGUUAAAAUAAGGCUAGUCCGUUAUCAACUUGAAAAAGUGGCACCGAGUCGGUGCUUUU-3’</td><td colspan="2"></td></tr>
<tr><td>gRNA-GFP+4</td><td>5’-GGGAUGCAUGCCGUUCUUCUGCUUGUGUUUUAGAGCUAGAAAUAGCAAGUUAAAAUAAGGCUAGUCCGUUAUCAACUUGAAAAAGUGGCACCGAGUCGGUGCUUUU-3’</td><td colspan="2"></td></tr>
<tr><td>gRNA-GFP+8</td><td>5’-GGGAUGCAUGCAUGCCGUUCUUCUGCUUGUGUUUUAGAGCUAGAAAUAGCAAGUUAAAAUAAGGCUAGUCCGUUAUCAACUUGAAAAAGUGGCACCGAGUCGGUGCUUUU-3’</td><td colspan="2"></td></tr>
<tr><td>gRNA-HBEGF</td><td>5’-GGGUUCUCUCGGCACUGGUGACGUUUUAGAGCUAGAAAUAGCAAGUUAAAAUAAGGCUAGUCCGUUAUCAACUUGAAAAAGUGGCACCGAGUCGGUGCUUUU-3’</td><td colspan="2"></td></tr>
<tr><td>gRNA-ANTXR1</td><td>5’-GGGAGCGGAGAGCCCUCGGCAUGUUUUAGAGCUAGAAAUAGCAAGUUAAAAUAAGGCUAGUCCGUUAUCAACUUGAAAAAGUGGCACCGAGUCGGUGCUUUU-3’</td><td colspan="2"></td></tr>
<tr><td>crGFP</td><td>5’-GGGAUGCCGUUCUUCUGCUUGUGUUUUAGAGCUAUGCUGUUUUG-3’</td><td colspan="2"></td></tr>
<tr><td>crHBEGF</td><td>5’-GGGUUCUCUCGGCACUGGUGACGUUUUAGAGCUAUGCUGUUUUG-3’</td><td colspan="2"></td></tr>
<tr><td>tracrRNA-F</td><td>5’-TCTAATACGACTCACTATAGGGTTGGAACCATTCAAAACAGCATAGCAAGTTAAAATAAGGCTAG-3’</td><td colspan="2" rowspan="2">For tracrRNA construct</td></tr>
<tr><td>tracrRNA-R</td><td>5’-AAAAAAAGCACCGACTCGGTGCCACTTTTTCAAGTTGATAACGGACTAGCCTTATTTTAACTTGCT-3’</td></tr>
<tr><td>tracrRNA</td><td>5’-GGGUUGGAACCAUUCAAAACAGCAUAGCAAGUUAA</td><td colspan="2"></td></tr>
</table>

	AAUAAGGCUAGUCCGUUAUCAACUUGAAAAAGUGG CACCGAGUCGGUGCUUUUUUU-3’	
t-GFP-1F	5’-GAGGAGCTGTTCACCGGG-3’	For PCR of t-GFP1
t-GFP-1R	5’-CTTGTACAGCTCGTCCATGC-3’	
t-GFP-2F	5’-GACGTAAACGGCCACAAGTTC-3’	For PCR of t-GFP2
t-GFP-2R	5’-GGGGTGTTCTGCTGGTAGTG-3’	
t-HBEGF-F	5’-GCCGCTTCGAAAGTGACTGG-3’	For PCR of t-HBEGF
t-HBEGF-R	5’-GATCCCCCAGTGCCCATCAG-3’	
t-ANTXR1-F	5’-AAGCGGAGGACAGGATTGGG-3’	For PCR of t-ANTXR1
t-ANTXR1-R	5’-CCTCTGTGGCCCTGGAGATG-3’	
t-OT1-HBEGF-F	5’-TTCTCCATGTTCCGCTAGCAC-3’	For PCR of t-OT1-HBEGF
t-OT1-HBEGF-R	5’-AACCCGCAACTCTGCCAAGA-3’	
t-OT2-HBEGF-F	5’-AGGACAATGGCGCTGTCTCC-3’	For PCR of t-OT2-HBEGF
t-OT2-HBEGF-R	5’-CACTCCCCTTGGCTCTCAGC-3’	
crRNA13a	5’-GAUUUAGACUACCCCAAAAACGAAGGGGACUAAAAC UAGAUUGCUGUUCUACCAAGUAAUCCAU-3’	
target13a	5’-FAM-UUACUUGGUAGAACAGCAAUCUA-3’	
reporter13a	5’-FAM-UUUUU-BHQ1-3’	
crRNA13a-Cy3	5’-Cy3-GAUUUAGACUACCCCAAAAACGAAGGGGACUAAAAC UAGAUUGCUGUUCUACCAAGUAAUCCAU-3’	
Protector-GFP	5’-ACACAA-*-GCAGAA-*-GAACGG-*-CATCCC-3’	* Photocleavable linker
Protector-HBEGF	5’-ACGTCA-*-CCAGTG-*-CCGAGA-*-GAACCC-3’	* Photocleavable linker

Supplementary Table 1. DNA and RNA sequences used in the current study

	Protein engineering of Cas9	Conditional expression of Cas9 or gRNA	Near-infrared upconversion-activated CRISPR system	Photocleavable protector-based regulation	The current method
Regulator	Small molecules or UV light	Small molecules	Near-infrared light	UV light	Small molecules
Requirements	Modification of specific amino acids in Cas9 backbone	Additional trans-acting factors and promoters to be incorporated in the construction of the delivery system	Covalent conjugation of Cas9 protein to upconverting nanoparticles via carbodiimide cross-linker chemistry	Complementary DNA protector containing photocleavable groups	Post-synthetic masking of gRNA
Pros & Cons	High effort to engineer or modify Cas9;<br>Labor intensive;<br>Highly customizable	Complicated cloning steps or multiple vectors;<br>Suffer from leaky expression	High effort to fabricate upconverting nanoparticles-Cas9@PEI;<br>Labor intensive;<br>Difficult to customize	Highly customizable;<br>Protect light-labile DNA during preparation and processing;<br>Cellular and DNA damage under prolonged UV exposure	Easily implemented method;<br>Improved stability of masked gRNA;<br>Highly customizable
Supplementary references	References[5-8]	References[9-12]	Reference[13]	Reference[4]	

Supplementary Table 2. Comparisons between the proposed method with existing approaches for regulating CRISPR activity

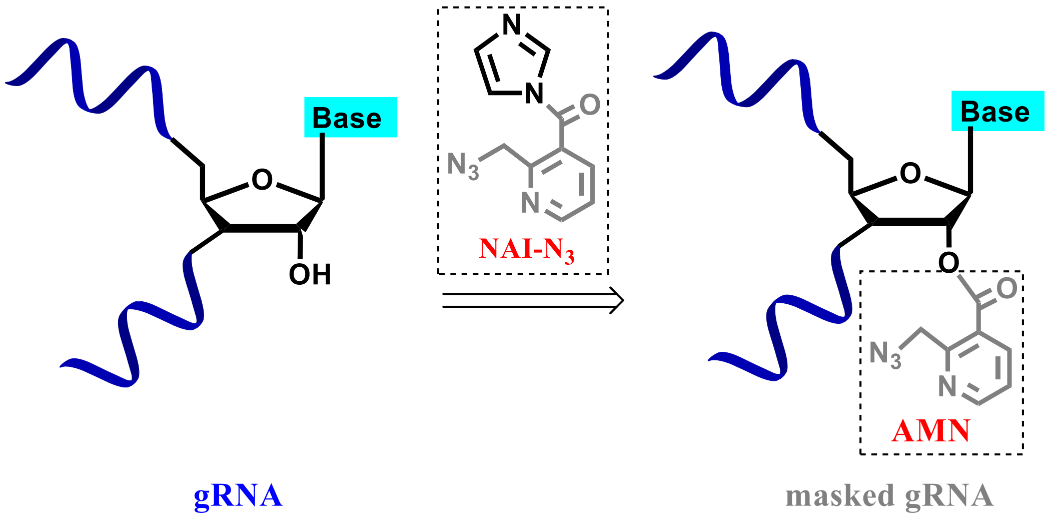

Supplementary Figure 1. Schematic illustration of the design and workflow. The post-synthetic chemistry was used to mask gRNA and further block CRISPR functions. An RNA structure profiling probe, 2-azidomethylnicotinic acid imidazolide (NAI-N_3), was used.

A

GAGGAGCTGTTCACCGGGGTGGTGCCCATCCTGGTCGAGCTGGACGGCGACGTAAA
CGGCCACAAGTTCAGCGTGTCCGGCGAGGGCGAGGGCGATGCCACCTACGGCAAGCT
GACCCTGAAGTTCATCTGCACCACCGGCAAGCTGCCCGTGCCCTGGCCCACCCTCGTG
ACCACCCTGACCTACGGCGTGCAGTGCTTCAGCCGCTACCCCGACCACATGAAGCAG
CACGACTTCTTCAAGTCCGCCATGCCCGAAGGCTACGTCCAGGAGCGCACCATCTTCT
TCAAGGACGACGGCAACTACAAGACCCGCGCCGAGGTGAAGTTCGAGGGCGACACC
CTGGTGAACCGCATCGAGCTGAAGGGCATCGACTTCAAGGAGGACGGCAACATCCTG
GGGCACAAGCTGGAGTACAACTACAACAGCCACAACGTCTATATCATGGCCG**ACAAG**
CAGAAGAACGGCA^TCAAGGTGAACTTCAAGATCCGCCACAACATCGAGGACGGCA
GCGTGCAGCTCGCCGACCACTACCAGCAGAACACCCCCATCGGCGACGGCCCCGTGC
TGCTGCCCGACAACCACTACCTGAGCACCCAGTCCGCCCTGAGCAAAGACCCCAACG
AGAAGCGCGATCACATGGTCCTGCTGGAGTTCGTGACCGCCGCCGGGATCACTCTCG
GCATGGACGAGCTGTACAAG

B

GACGTAAACGGCCACAAGTTCAGCGTGTCCGGCGAGGGCGAGGGCGATGCCACCTA
CGGCAAGCTGACCCTGAAGTTCATCTGCACCACCGGCAAGCTGCCCGTGCCCTGGCCC
ACCCTCGTGACCACCCTGACCTACGGCGTGCAGTGCTTCAGCCGCTACCCCGACCACA
TGAAGCAGCACGACTTCTTCAAGTCCGCCATGCCCGAAGGCTACGTCCAGGAGCGCA
CCATCTTCTTCAAGGACGACGGCAACTACAAGACCCGCGCCGAGGTGAAGTTCGAGG
GCGACACCCTGGTGAACCGCATCGAGCTGAAGGGCATCGACTTCAAGGAGGACGGCA
ACATCCTGGGGCACAAGCTGGAGTACAACTACAACAGCCACAACGTCTATATCATGG
CCG**ACAAGCAGAAGAACGGCA^TC**AAGGTGAACTTCAAGATCCGCCACAACATCGA
GGACGGCAGCGTGCAGCTCGCCGAC**CACTACCAGCAGAACACCCC**

Supplementary Figure 2. Schematic illustration of GFP sequences around target loci. The target loci and PCR primer loci were indicated by blue color and underlining, respectively. Red caret showed the cleavage sites by Cas9 nuclease. PCR primers flanking the target regions were designed by Primer 3 software. We generated two different GFP targets (t-GFP1 and t-GFP2) carrying the target loci from pEGFP-C1 vector. (**A**) The t-GFP1 was demonstrated. (**B**) The t-GFP2 was demonstrated.

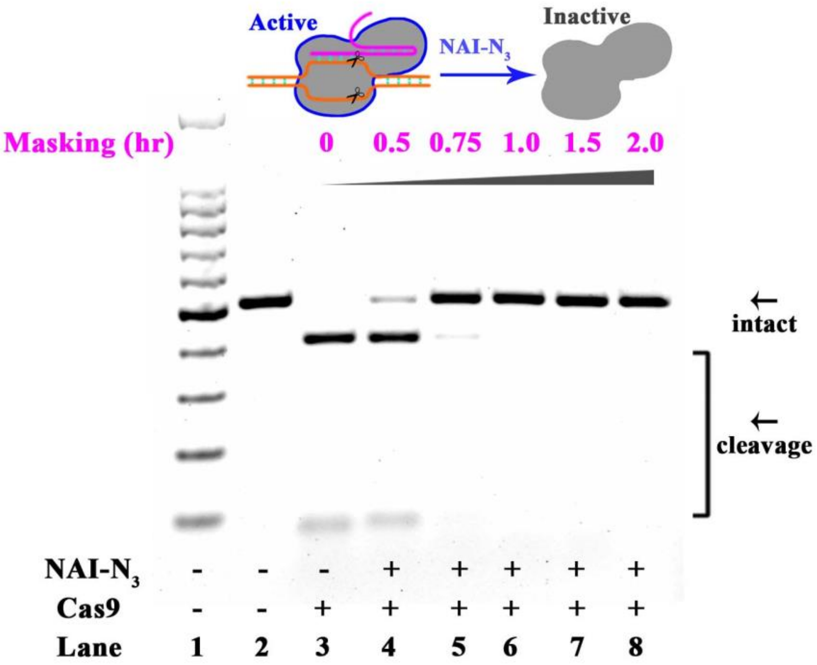

Supplementary Figure 3. Conditional control of RNA-guided DNA cleavage. The influence of chemical masking of gRNA on Cas9 cleavage of t-GFP2. Reactions were carried out as described in the Experimental Section. The gRNA (gRNA-GFP) was synthesized by *in vitro* transcription with T7 RNA polymerase. Uncleaved t-GFP2 DNA (500 bp) cut to shorter cleavage fragments (421 bp and 79 bp) were indicated. Lane 1: DNA markers; lane 2: no Cas9 control; lane 3: original gRNA-GFP; lanes 4 - 8: gRNA-GFP with different acylation levels.

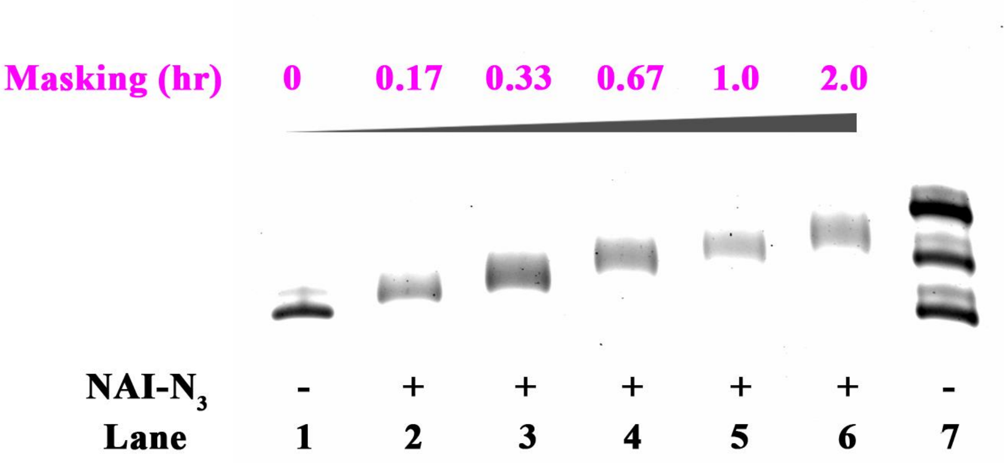

Supplementary Figure 4. The study of the extent of gRNA acylation. A denaturing electrophoresis was performed to examine the reaction products. The gRNAs (gRNA-GFP, gRNA-GFP+4 and gRNA-GFP+8) were synthesized by *in vitro* transcription with T7 RNA polymerase. The ribose 2'-OH acylation is accompanied by a 160-Da MW (molecular weight) increment corresponding to one AMN adduct, which means that the MW of two AMN adducts was roughly equal to the mean MW of one nucleobase (320 Da). For a 1-hour masking, up to eight AMN adducts were clearly recognizable by comparing the sample band to the RNA markers. Lane 1: original gRNA-GFP; lanes 2 - 6: gRNA-GFP with different acylation levels; lane 7: RNA markers (gRNA-GFP, gRNA-GFP+4 and gRNA-GFP+8).

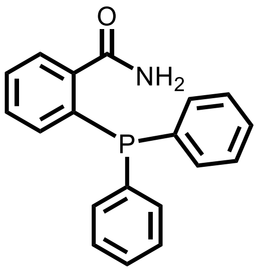

2-(diphenylphosphanyl)benzamide
(DPBM)

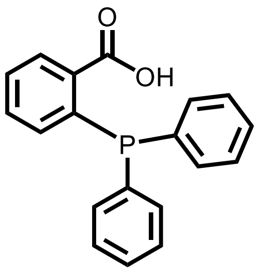

2-(diphenylphosphino)benzoic acid
(DPBA)

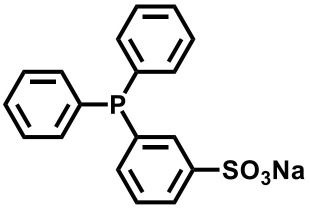

Sodium diphenylphosphinobenzene-3-sulfonate
(DPBS)

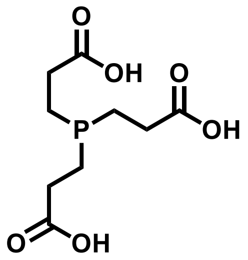

tris(2-carboxyl)phosphine
(TCEP)

Supplementary Figure 5. Chemical structure of the used phosphines. Chemical structures of DPBM, DPBA, DPBS and TCEP were demonstrated here.

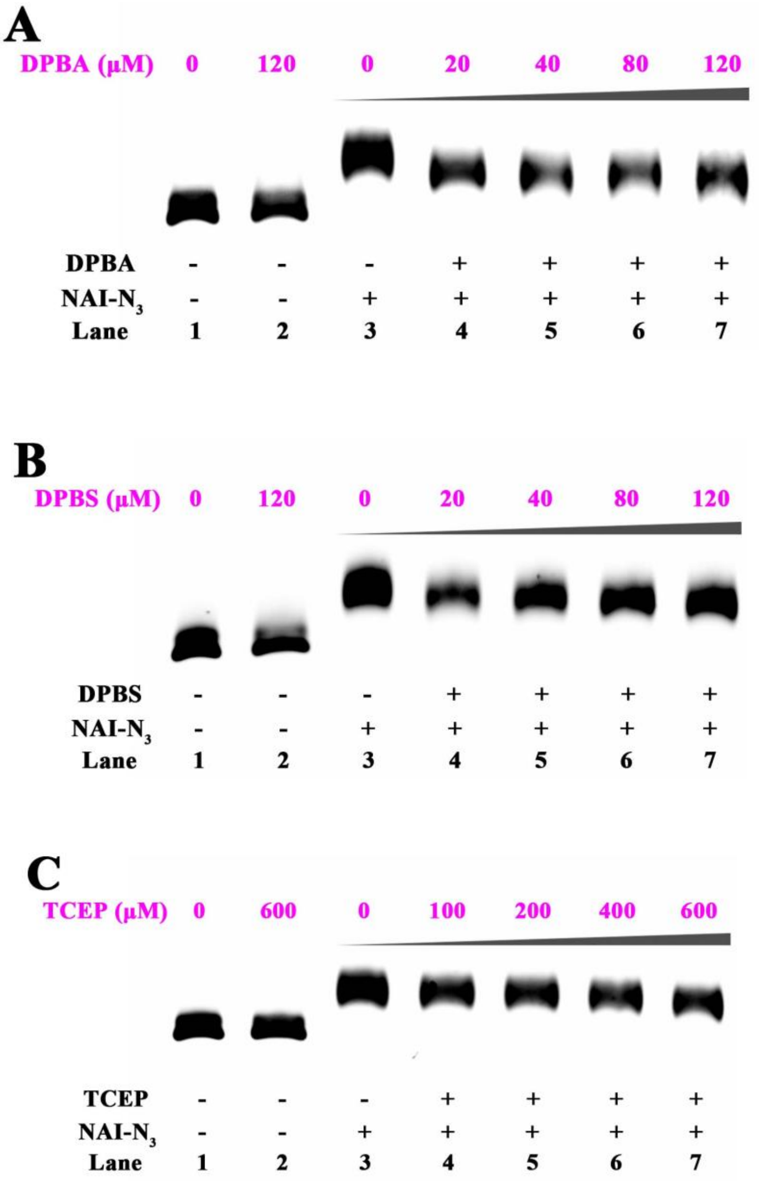

Supplementary Figure 6. Staudinger reduction drives the removal of AMN groups. Reactions were carried out as described in the Experimental Section. Image of representative data was shown here. The un-masking of acylated gRNA was confirmed by the appearance of faster-migrating bands, compared with the acylated sample. (**A**) The concentration-dependent removal of AMN groups with DPBA. Lanes 1: original gRNA-GFP; lane 2: original gRNA-GFP, 120 μM DPBA; lanes 3 - 7: acylated gRNA-GFP (200 mM NAI-N_3, 2.0 hr), different concentrations of DPBA. (**B**) The concentration-dependent removal of AMN groups with DPBS. Lanes 1: original gRNA-GFP; lane 2: original gRNA-GFP, 120 μM DPBS; lanes 3 - 7: acylated gRNA-GFP (200 mM NAI-N_3, 2.0 hr), different concentrations of DPBS. (**C**) The treatment of gRNA-GFP with TCEP. Lanes 1: original gRNA-GFP; lane 2: original gRNA-GFP, 600 μM DPBM; lanes 3 - 7: acylated gRNA-GFP (200 mM NAI-N_3, 2.0 hr), different concentrations of TCEP.

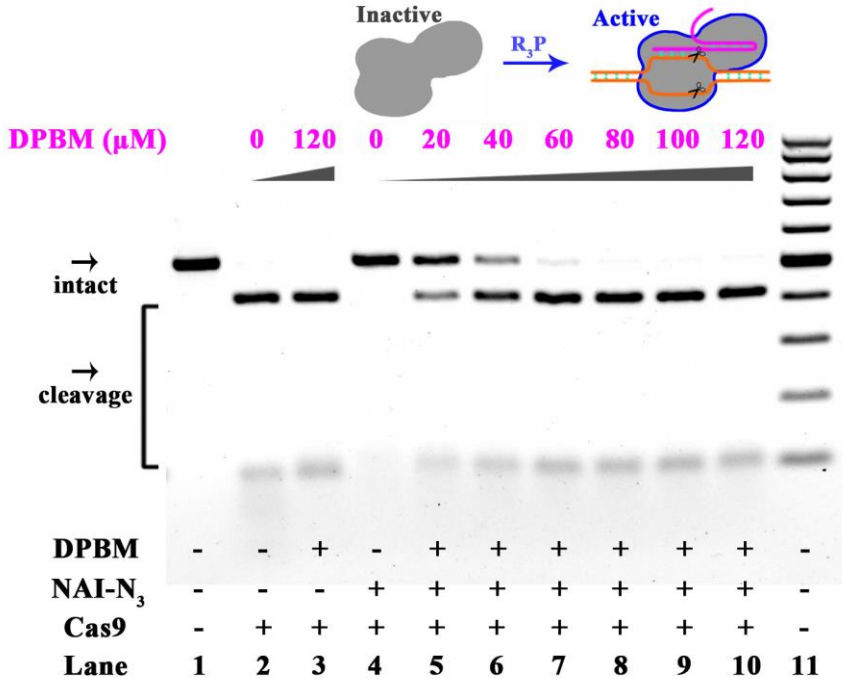

Supplementary Figure 7. Conditional control of RNA-guided DNA cleavage. The influence of DPBM on Cas9 cleavage of t-GFP2 was shown here. The CRISPR/Cas9 system with acylated gRNA-GFP (200 mM $NAI-N_3$, 2 hr) was treated with various concentrations of DPBM ranging from 0 to 120 μM. Reactions were carried out as described in the Experimental Section. Image of representative gel was shown here. Uncleaved t-GFP2 DNA (500 bp) cut to shorter cleavage fragments (421 bp and 79 bp) were indicated. Lanes 1: no Cas9 control; lane 2: original gRNA-GFP; lane 3: original gRNA-GFP, 120 μM DPBM; lanes 4 - 10: acylated gRNA-GFP (200 mM $NAI-N_3$, 2.0 hr), different concentrations of DPBM; lane 11: DNA markers.

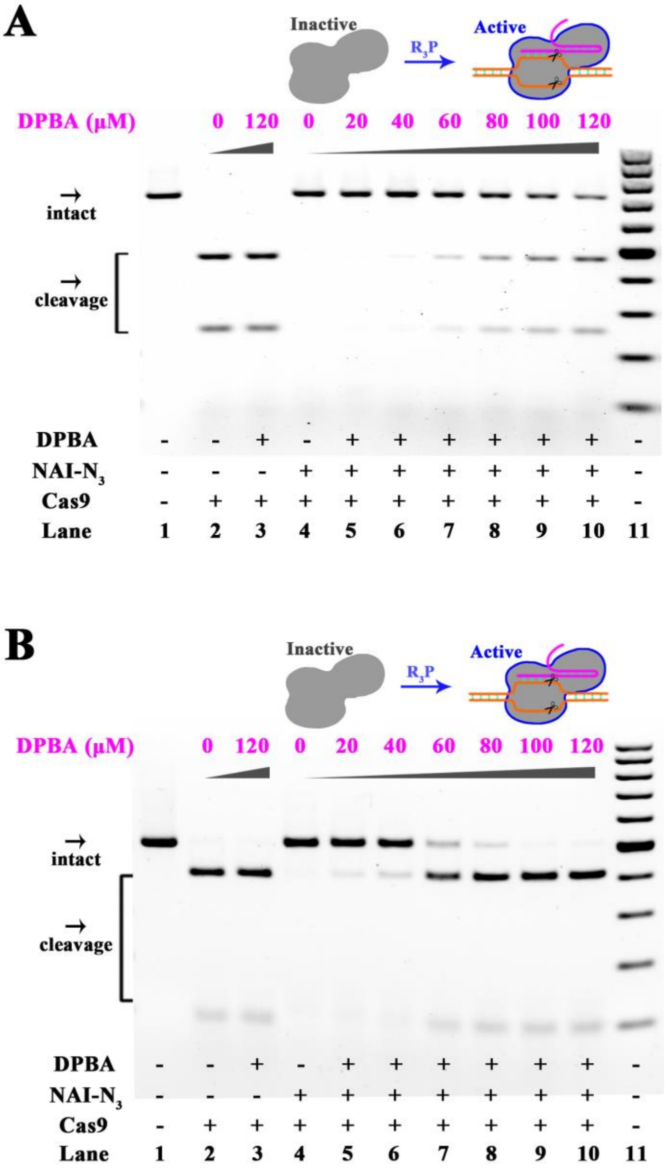

Supplementary Figure 8. Conditional control of RNA-guided DNA cleavage. The DPBA is used in the fabrication of CrCS. Reactions were carried out as described in the Experimental Section. The CRISPR/Cas9 system with acylated gRNA-GFP (200 mM NAI-N_3, 2 hr) was treated with various concentrations of DPBA ranging from 0 to 120 μM. Representative gel images were demonstrated here. Lanes 1: no Cas9 control; lane 2: original gRNA-GFP; lane 3: original gRNA-GFP, 120 μM DPBA; lanes 4 - 10: acylated gRNA-GFP (200 mM NAI-N_3, 2.0 hr), different concentrations of DPBA; lane 11: DNA markers. (**A**) The t-GFP1 was used as the double-stranded DNA substrate for Cas9 cleavage. Uncleaved t-GFP1 DNA (702 bp) cut to shorter cleavage fragments (469 bp and 233 bp) were indicated. (**B**) The t-GFP2 was used as the double-stranded DNA substrate for Cas9 cleavage. Uncleaved t-GFP2 DNA (500 bp) cut to shorter cleavage fragments (421 bp and 79 bp) were indicated.

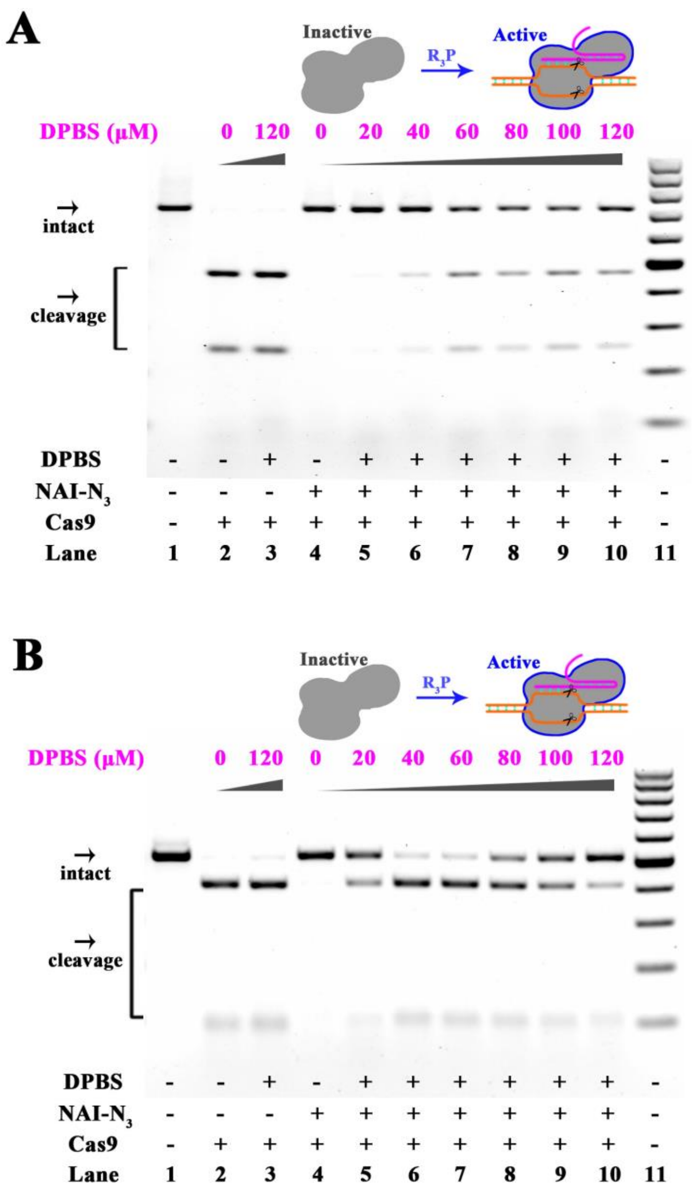

Supplementary Figure 9. Conditional control of RNA-guided DNA cleavage. The DPBS was used in the fabrication of CrCS. Reactions were carried out as described in the Experimental Section. The CRISPR/Cas9 system with acylated gRNA-GFP (200 mM NAI-N_3, 2 hr) was treated with various concentrations of DPBS ranging from 0 to 120 µM. Representative gel images were demonstrated here. Lanes 1: no Cas9 control; lane 2: original gRNA-GFP; lane 3: original gRNA-GFP, 120 µM DPBS; lanes 4 - 10: acylated gRNA-GFP (200 mM NAI-N_3, 2.0 hr), different concentrations of DPBS; lane 11: DNA markers. (**A**) The t-GFP1 was used as the double-stranded DNA substrate for Cas9 cleavage. Uncleaved t-GFP1 DNA (702 bp) cut to shorter cleavage fragments (469 bp and 233 bp) were indicated. (**B**) The t-GFP2 was used as the double-stranded DNA substrate for Cas9 cleavage. Uncleaved t-GFP2 DNA (500 bp) cut to shorter cleavage fragments (421 bp and 79 bp) were indicated.

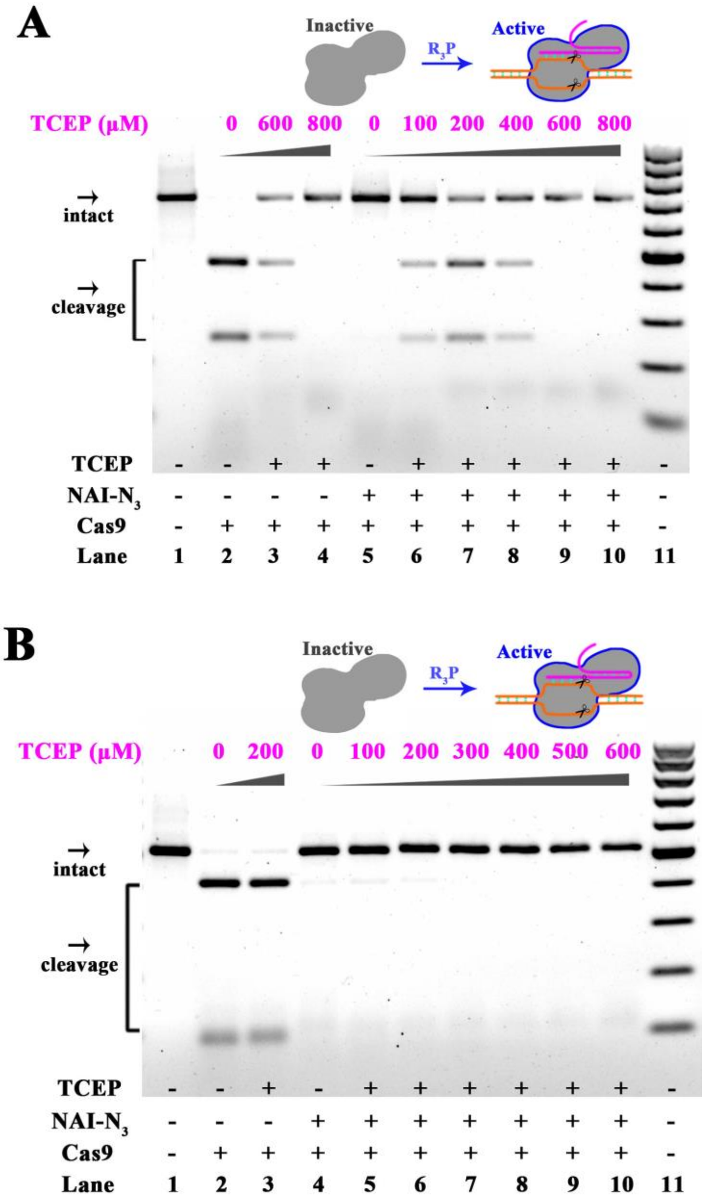

Supplementary Figure 10. The influence of TCEP on Cas9 cleavage. Reactions were carried out as described in the Experimental Section. The CRISPR/Cas9 system with acylated gRNA-GFP (200 mM NAI-N_3, 2 hr) was treated with various concentrations of TCEP. Representative gel images were demonstrated here. (**A**) The t-GFP1 was used as the double-stranded DNA substrate for Cas9 cleavage. Lanes 1: no Cas9 control; lane 2: original gRNA-GFP; lane 3: original gRNA-GFP, 600 μM TCEP; lane 4: original gRNA-GFP, 800 μM TCEP; lanes 5 - 10: acylated gRNA-GFP (200 mM NAI-N_3, 2.0 hr), different concentrations of TCEP; lane 11: DNA markers. (**B**) The t-GFP2 was used as the double-stranded DNA substrate for Cas9 cleavage. Lanes 1: no Cas9 control; lane 2: original gRNA-GFP; lane 3: original gRNA-GFP, 200 μM TCEP; lanes 4 - 10: acylated gRNA-GFP (200 mM NAI-N_3, 2.0 hr), different concentrations of TCEP; lane 11: DNA markers.

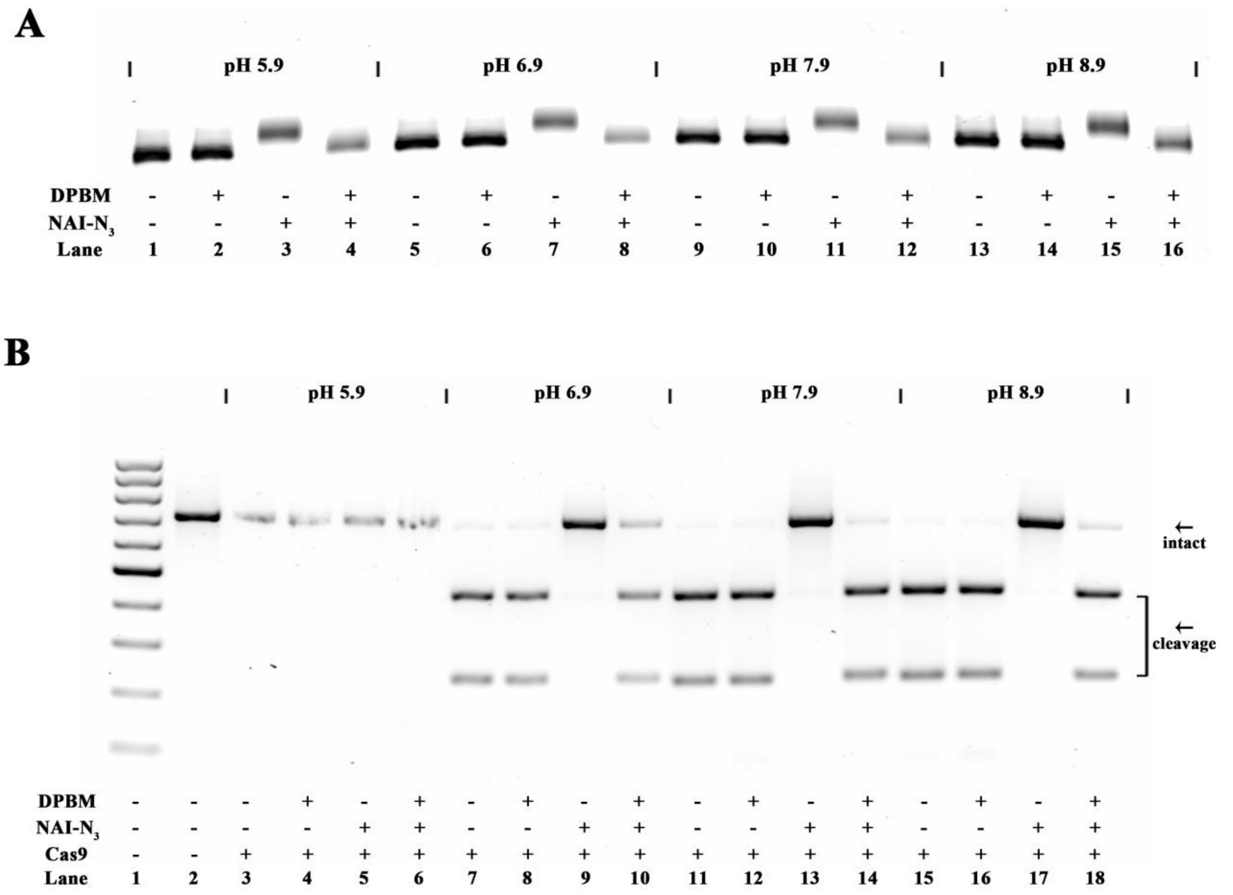

Supplementary Figure 11. The pH effects on the Staudinger reduction. Reactions were carried out as described in the Experimental Section. Representative gel images are demonstrated here. (**A**) A denaturing electrophoresis was performed to examine the reaction products. Lane 1: original gRNA-GFP; lane 2: original gRNA-GFP, 120 μM DPBM; lane 3: acylated gRNA-GFP (200 mM NAI-N_3, 2.0 hr); lane 4: acylated gRNA-GFP (200 mM NAI-N_3, 2.0 hr), 120 μM DPBM; lane 5: original gRNA-GFP; lane 6: original gRNA-GFP, 120 μM DPBM; lane 7: acylated gRNA-GFP (200 mM NAI-N_3, 2.0 hr); lane 8: acylated gRNA-GFP (200 mM NAI-N_3, 2.0 hr), 120 μM DPBM; lane 9: original gRNA-GFP; lane 10: original gRNA-GFP, 120 μM DPBM; lane 11: acylated gRNA-GFP (200 mM NAI-N_3, 2.0 hr); lane 12: acylated gRNA-GFP (200 mM NAI-N_3, 2.0 hr), 120 μM DPBM; lane 13: original gRNA-GFP; lane 14: original gRNA-GFP, 120 μM DPBM; lane 15: acylated gRNA-GFP (200 mM NAI-N_3, 2.0 hr); lane 16: acylated gRNA-GFP (200 mM NAI-N_3, 2.0 hr), 120 μM DPBM. Lanes 1-4: pH = 5.9; lanes 5-8: pH = 6.9; lanes 9-12: pH = 7.9; lanes 13-16: pH = 8.9. (**B**) The CRISPR/Cas9 system was examined in the reaction buffer (50 mM Tris-HCl, 100 mM NaCl, 10 mM $MgCl_2$ and 100 μg per mL BSA) at different pH ranging from 5.9 to 8.9. The t-GFP1 was used as the double-stranded DNA substrate for Cas9 cleavage. Uncleaved t-GFP1 DNA (702 bp) cut to shorter cleavage fragments (469 bp and 233 bp) were indicated. Lane 1: DNA markers; lane 2: no Cas9 control; lanes 3-6: pH = 5.9; lanes 7-10: pH = 6.9; lanes 11-14: pH = 7.9; lanes 15-18: pH = 8.9.

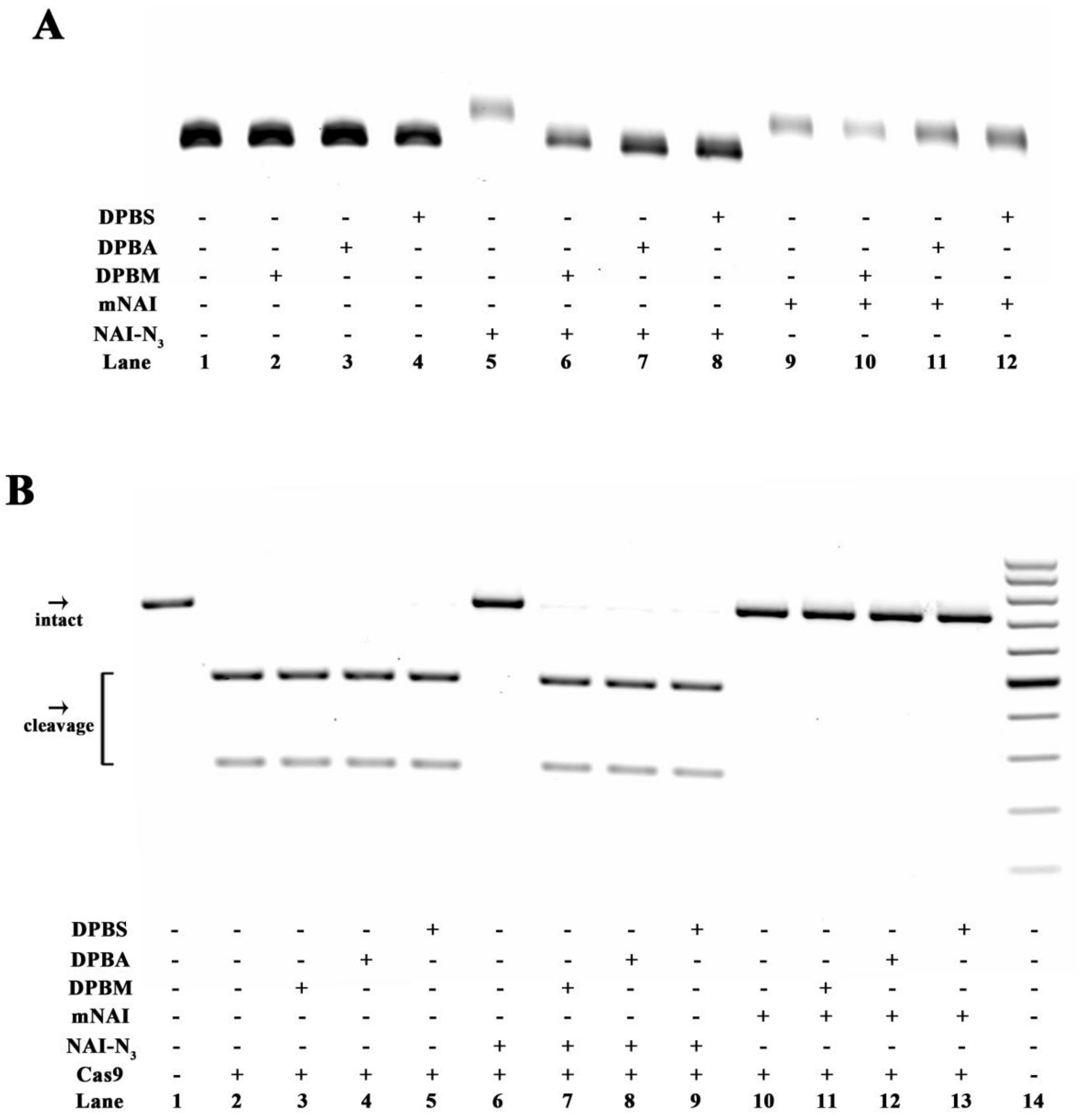

Supplementary Figure 12. The examination of spontaneous unmasking of gRNA adducts. Reactions were carried out as described in the Experimental Section. Representative gel images were demonstrated here. (**A**) The reactivity of mNAI toward 2'-OH groups in RNA ribose. Lane 1: original gRNA-GFP; lane 2: original gRNA-GFP, 120 μM DPBM; lane 3: original gRNA-GFP, 120 μM DPBA; lane 4: original gRNA-GFP, 120 μM DPBS; lane 5: the NAI-N_3-treated gRNA-GFP; lane 6: the NAI-N_3-treated gRNA-GFP, 120 μM DPBM; lane 7: the NAI-N_3-treated gRNA-GFP, 120 μM DPBA; lane 8: the NAI-N_3-treated gRNA-GFP, 120 μM DPBS; lane 9: the mNAI-treated gRNA-GFP; lane 10: the mNAI-treated gRNA-GFP, 120 μM DPBM; lane 11: the mNAI-treated gRNA-GFP, 120 μM DPBA; lane 12: the mNAI-treated gRNA-GFP, 120 μM DPBS. (**B**) The t-GFP1 was used as the double-stranded DNA substrate for Cas9 cleavage. Uncleaved t-GFP1 DNA (702 bp) cut to shorter cleavage fragments (469 bp and 233 bp) were indicated.

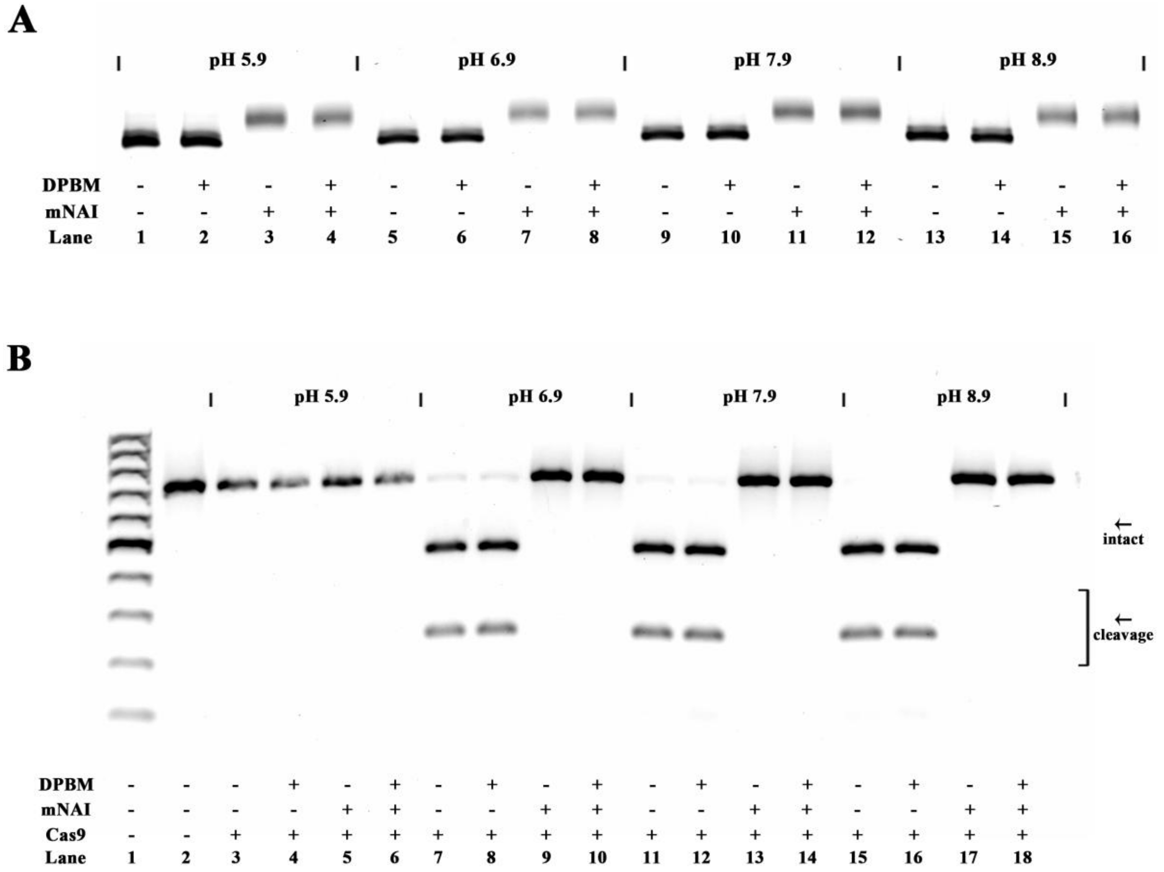

Supplementary Figure 13. The examination of spontaneous unmasking of gRNA adducts with various pH conditions. Reactions were carried out as described in the Experimental Section. Representative gel images were demonstrated here. (**A**) A denaturing electrophoresis was performed to examine the reaction products. Lane 1: original gRNA-GFP; lane 2: original gRNA-GFP, 120 μM DPBM; lane 3: acylated gRNA-GFP (200 mM mNAI, 2.0 hr); lane 4: acylated gRNA-GFP (200 mM mNAI, 2.0 hr), 120 μM DPBM; lane 5: original gRNA-GFP; lane 6: original gRNA-GFP, 120 μM DPBM; lane 7: acylated gRNA-GFP (200 mM mNAI, 2.0 hr); lane 8: acylated gRNA-GFP (200 mM mNAI, 2.0 hr), 120 μM DPBM; lane 9: original gRNA-GFP; lane 10: original gRNA-GFP, 120 μM DPBM; lane 11: acylated gRNA-GFP (200 mM mNAI, 2.0 hr); lane 12: acylated gRNA-GFP (200 mM mNAI, 2.0 hr), 120 μM DPBM; lane 13: original gRNA-GFP; lane 14: original gRNA-GFP, 120 μM DPBM; lane 15: acylated gRNA-GFP (200 mM mNAI, 2.0 hr); lane 16: acylated gRNA-GFP (200 mM mNAI, 2.0 hr), 120 μM DPBM. Lanes 1-4: pH = 5.9; lanes 5-8: pH = 6.9; lanes 9-12: pH = 7.9; lanes 13-16: pH = 8.9. (**B**) The CRISPR/Cas9 system was examined in the reaction buffer (50 mM Tris-HCl, 100 mM NaCl, 10 mM $MgCl_2$ and 100 μg per mL BSA) at different pH ranging from 5.9 to 8.9. The t-GFP1 was used as the double-stranded DNA substrate for Cas9 cleavage. Uncleaved t-GFP1 DNA (702 bp) cut to shorter cleavage fragments (469 bp and 233 bp) were indicated. Lane 1: DNA markers; lane 2: no Cas9 control; lanes 3-6: pH = 5.9; lanes 7-10: pH = 6.9; lanes 11-14: pH = 7.9; lanes 15-18: pH = 8.9.

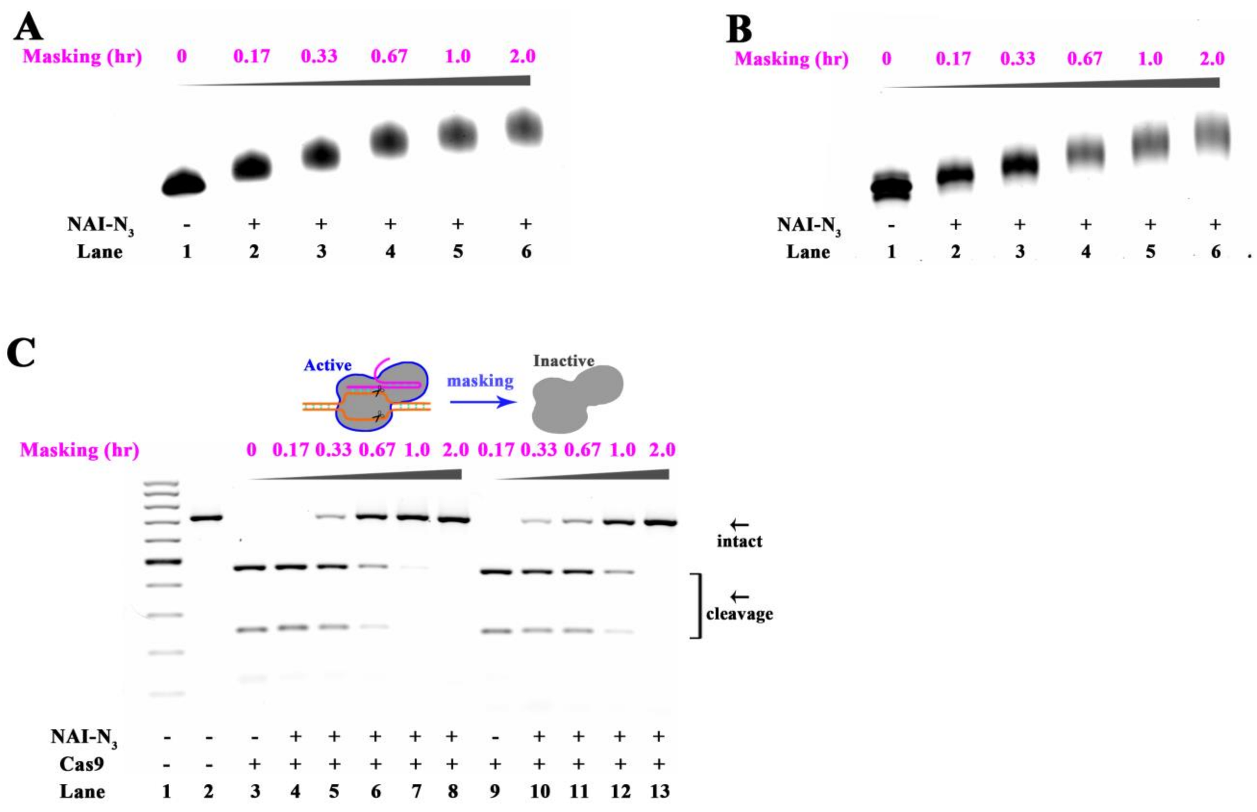

Supplementary Figure 14. **Conditional control of RNA-guided DNA cleavage by individually masking the crRNA and tracrRNA components.** Reactions were carried out as described in the Experimental Section. Image of representative data was shown here. (**A**) The masking of crGFP. (**B**) The masking of tracrRNA. The tracrRNA was synthesized by *in vitro* transcription with T7 RNA polymerase. For (**A**) and (**B**), the GelRed-stained RNA was visualized using a Pharos FX Molecular imager (Bio-Rad, USA) in the fluorescence mode (λex = 590 nm). (**C**) The influence of chemical masking on Cas9 cleavage of target GFP DNA. Uncleaved GFP DNA (702 bp) cut to shorter cleavage fragments (469 bp and 233 bp) were indicated. Lane 1: DNA markers; lane 2: no Cas9 control; lanes 3: original tracrRNA + original crGFP; lanes 4-8: original tracrRNA + acylated crGFP (different masking levels); lanes 9 - 13: original crGFP + acylated tracrRNA (different masking levels).

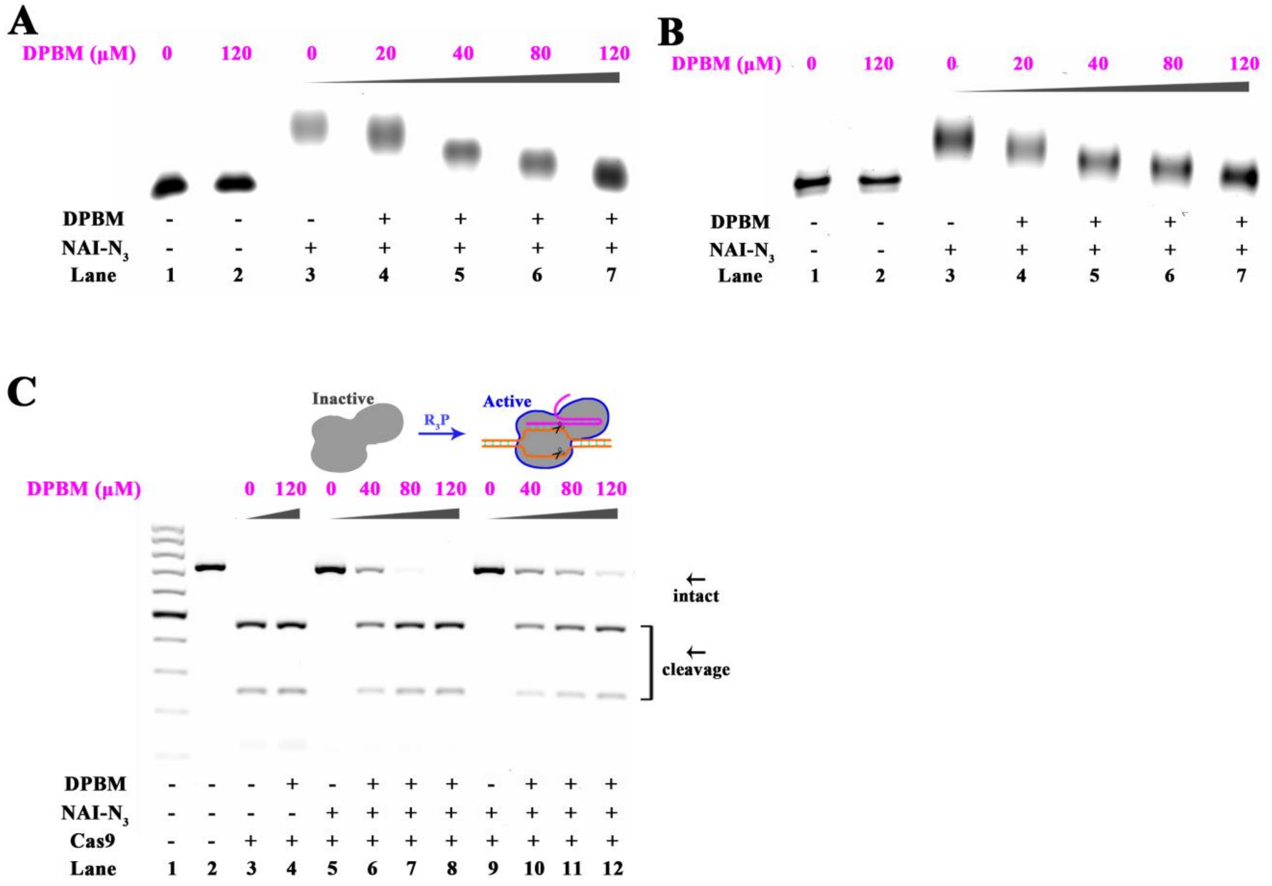

Supplementary Figure 15. **Conditional control of RNA-guided DNA cleavage by individually manipulating the crRNA and tracrRNA components.** Reactions were carried out as described in the Experimental Section. Image of representative data was shown here. (**A**) The unmasking of the acylated crGFP. Lanes 1: original crGFP; lane 2: original crGFP, 120 μM DPBM; lanes 3 - 7: acylated crGFP (200 mM NAI-N_3, 2.0 hr), different concentrations of DPBM. (**B**) The unmasking of the acylated tracrRNA. Lanes 1: original tracrRNA; lane 2: original tracrRNA, 120 μM DPBM; lanes 3 - 7: acylated tracrRNA (200 mM NAI-N_3, 2.0 hr), different concentrations of DPBM. For (**A**) and (**B**), the GelRed-stained RNA was visualized using a Pharos FX Molecular imager (Bio-Rad, USA) in the fluorescence mode (λex = 590 nm). The AMN groups were removed by DPBM with a concentration-dependent manner. (**C**) The influence of chemical unmasking on Cas9 cleavage of target GFP DNA. Uncleaved GFP DNA (702 bp) cut to shorter cleavage fragments (469 bp and 233 bp) were indicated. Lane 1: DNA markers; lane 2: no Cas9 control; lane 3: original crGFP + original tracrRNA; lane 4: original crGFP + original tracrRNA, 120 μM DPBM; lanes 5-8: acylated crGFP (200 mM NAI-N_3, 2.0 hr) + original tracrRNA, different concentrations of DPBM; lanes 9-12: original crGFP + acylated tracrRNA (200 mM NAI-N_3, 2.0 hr), different concentrations of DPBM.

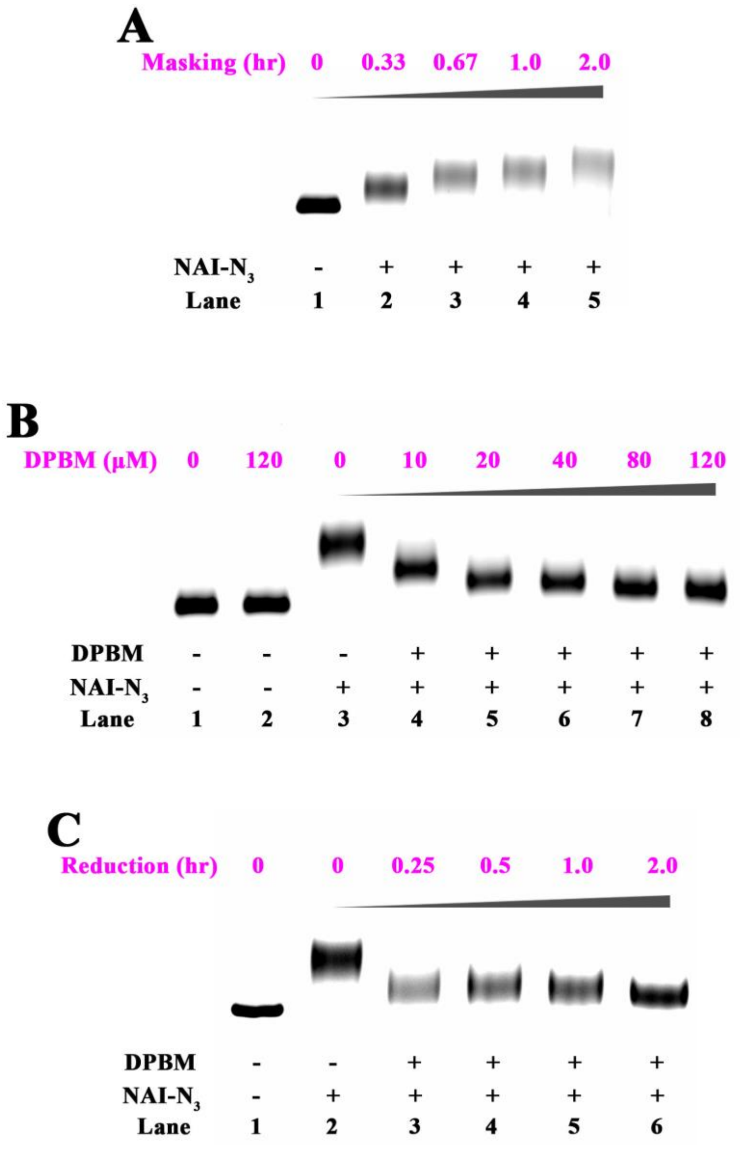

Supplementary Figure 16. Chemical masking and un-masking of crRNA13a. Staudinger reduction drives the removal of AMN groups. This assay was performed as described in the Experimental Section. After electrophoresis, crRNA13a was visualized using a Pharos FX Molecular imager (Bio-Rad, USA) in the fluorescence mode (λex = 590 nm). Representative data are demonstrated here. (**A**) In this assay, the more slowly migrating bands represent the higher-molecular weight crRNAs with acylation. (**B**) The AMN groups were removed by DPBM with a concentration-dependent manner. The unmasking of crRNA13a was confirmed by the appearance of faster-migrating bands, compared with the acylated sample. Lanes 1: original crRNA13a; lane 2: original crRNA13a, 120 μM DPBM; lanes 3 - 8: acylated crRNA13a (200 mM NAI-N_3, 2.0 hr), different concentrations of DPBM. (**C**) The AMN groups were removed by DPBM with a time-dependent manner. Lanes 1: original crRNA13a; lanes 2 - 6: acylated crRNA13a (200 mM NAI-N_3, 2.0 hr), the 120 μM DPBM treatment with different durations.

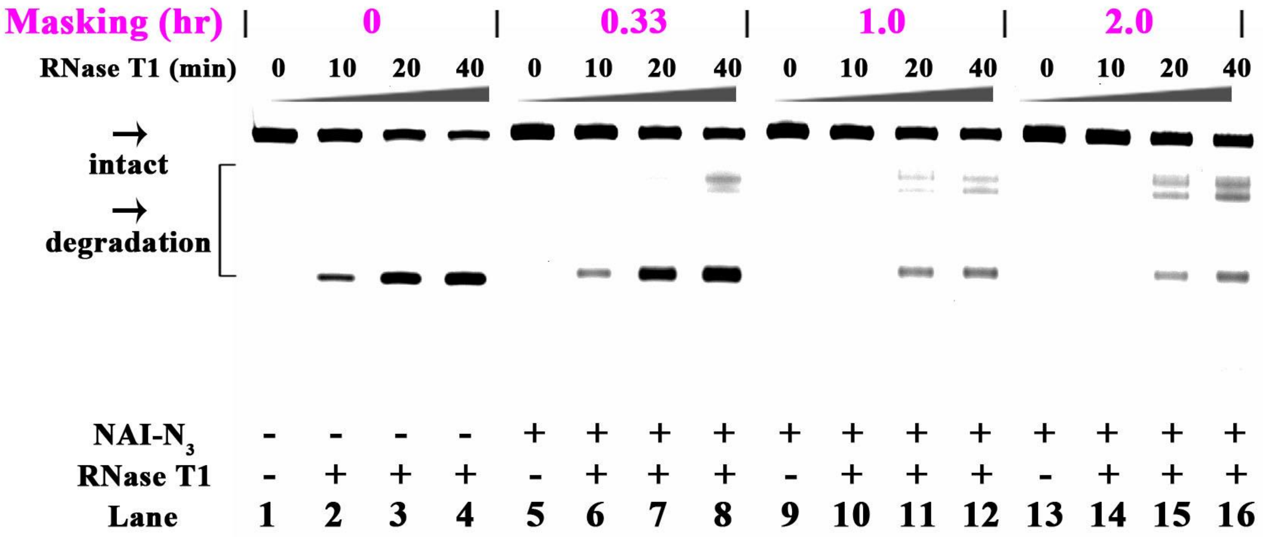

Supplementary Figure 17. Chemical masking protects gRNA from RNase degradation. Reactions were performed as described in the Experimental Section. All samples were tested in three biological replicates. Image of representative data was shown here. Lanes 1, 5, 9, 13: no RNase T1 control; lanes 2, 6, 10, 14: RNase T1 cleavage for 10 min; lanes 3, 7, 11, 15: RNase T1 cleavage for 20 min; lanes 4, 8, 12, 16: RNase T1 cleavage for 40 min.

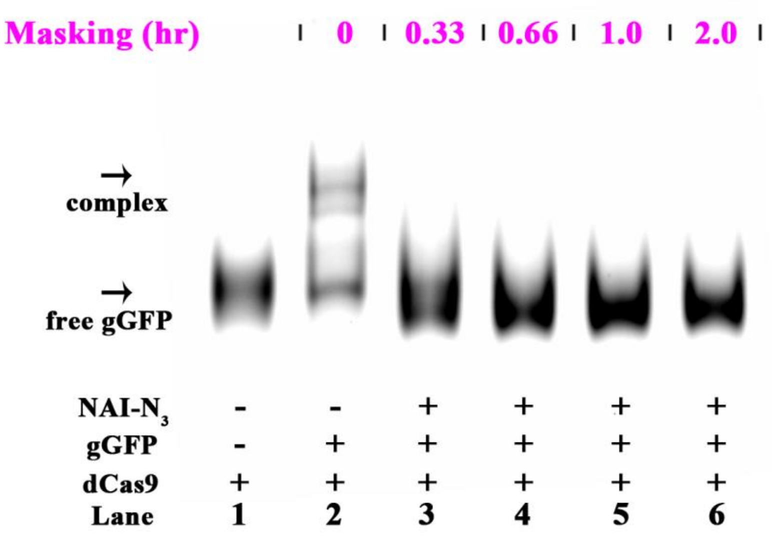

Supplementary Figure 18. Blocking the ability of gRNA to bind dCas9. Reactions were performed as described in the Experimental Section. All samples were tested in three biological replicates. Image of representative data was shown here. The fluorescently labeled dCas9 was incubated with gRNA-GFP with different treatments, and the samples were separated on native 6% PAGE gels. Lane 1: no gRNA control; lanes 2 - 6: gRNA-GFP with different masking levels was incubated with a 5.0 molar excess of dCas9 protein.

>gi|74230048|gb|CH471062.2|:13429990-13430610 Homo sapiens 211000035832302 genomic scaffold, whole genome shotgun sequence

GCCGCTTCGAAAGTGACTGGTGCCTCGCCGCCTCCTCTCGGTGCGGGACCATGAAGCT
GCTGCCGTCGGTGGTGCTGAAGCTCTTTCTGGCTGCAGGTAAGAGGGCTGCCGACGCC
CCCGGAGATCGGGGGGATGGGGGCGTTGTGCTGGGGGCATGGGGGAAGGTCGCCGC
AGCGCACCCGGCACGGGCCACTTGGTGGGGCCCTTGCGCTCTGGCGGACGGGCGTCG
GCATCGGTGCGTGTTGGTCAGGGGTCTGGGCGGGTGTCTGATGCGGCCTGGCCTCTCG
CCCGCAG**TTCTCTCGGCACTGGT**^**GAC**TGGCGAGAGCCTGGAGCGGCTTCGGAGAG
GGCTAGCTGCTGGAACCAGCAACCCGGACCCTCCCACTGTATCCACGGACCAGCTGC
TACCCCTAGGAGGCGGCCGGGACCGGAAAGTCCGTGACTTGCAAGAGGCAGATCTGG
ACCTTTTGAGAGGTGGGTGTGGAGGCCCCCCATCCTTGGACCTTGGTGGGCTGTTGAA
GAATAAGCAGATCCAAGATTCTTGCTGTTTGGGCAATACTGTGGGTTGAGGGTATTCA
TGGAGAACCTCGGGGAAAAGCTGATCGGCCTGATGGGCACTGGGGGATC

Supplementary Figure 19. Schematic illustration of the 5’-UTR sequence of *HBEGF* gene around target loci. The target loci and PCR primer loci were indicated by blue color and underlining, respectively. Red caret showed the cleavage sites by Cas9 nuclease. PCR primers flanking the target regions were designed by Primer 3 software. We generated target HBEGF DNA (t-HBEGF) carrying the target loci from HeLa-OC genomic DNA. The 19-nt sequence was the exact same sequence as the target sequence.

>NC_000002.12:69013265-69013771 Homo sapiens chromosome 2, GRCh38.p12 Primary Assembly

<u>AAGCGGAGGACAGGATTGGG</u>ATCCTTGAAACCCGAAACCCAGAAACAGCATCGGAG
CGGAAACCAGAGGGGAAACCTTGAACTCCTCCAGACAATTGCTTCCGGGGAGTTGCG
AGGGAGCGAGGGGGAATAAAGGACCCGCGAGGAAGGGCCCGCGGATGGCGCGTCCC
TGAGGGTCGTGGCGAGTTCGCGGAGCGTGGGAAGGAGCGGACCCTGCTCTCCCCGGG
CTGCGGGCCATGGCCACGGCGG**AGCGGAGAGCCCTCGG**^**CAT**CGGCTTCCAGTGGC
TCTCTTTGGCCACTCTGGTGCTCATCTGCGCCGGGCAAGGGGGACGCAGGGAGGATG
GGGGTCCAGCCTGCTACGGCGGATTTGACCTGTACTTCATTTTGGACAAGTAAGTGCC
GCGAGTTGTCCCCCCCACCCCAGGCTAAGCGGGCGAAAACGCTTTCGCCCCGGGCCG
GGCTCGTTGGCAGGGTCGCCTGGGGACAGGAGCG<u>CATCTCCAGGGCCACAGAGG</u>

Supplementary Figure 20. Schematic illustration of the 5’-UTR sequence of *ANTXR1* gene around target loci. The target loci and PCR primer loci were indicated by blue color and underlining, respectively. Red caret showed the cleavage sites by Cas9 nuclease. PCR primers flanking the target regions were designed by Primer 3 software. We generated target ANTXR1 DNA (t-ANTXR1) carrying the target loci from HeLa-OC genomic DNA. The 19-nt sequence was the exact same sequence as the target sequence.

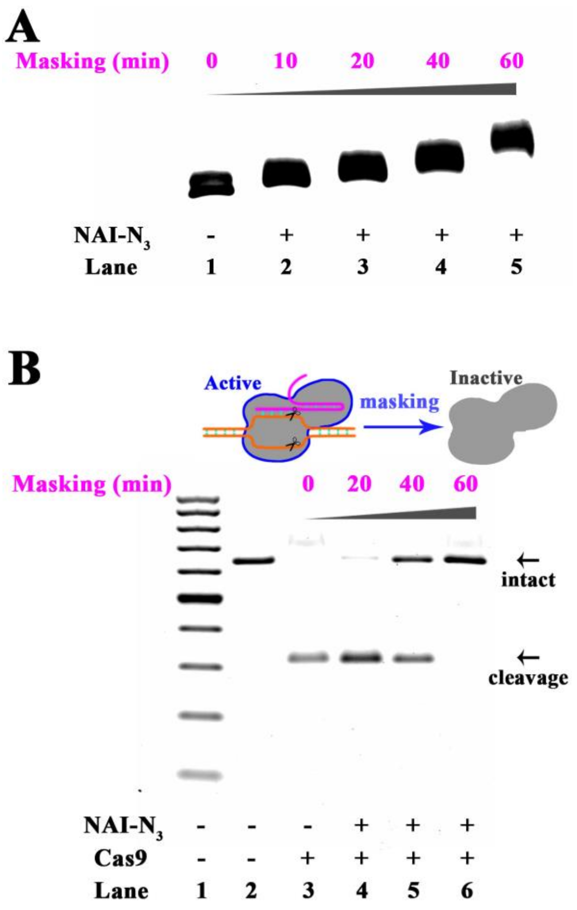

Supplementary Figure 21. **Conditional control of RNA-guided DNA cleavage.** Reactions were carried out as described in the Experimental Section. Image of representative data was shown here. (**A**) The gRNA (gRNA-HBEGF) was synthesized by *in vitro* transcription with T7 RNA polymerase. After electrophoresis, the GelRed-stained gRNA-HBEGF was visualized using a Pharos FX Molecular imager (Bio-Rad, USA) in the fluorescence mode (λex = 590 nm). In this assay, the more slowly migrating bands represented the higher-molecular weight gRNAs with acylation. (**B**) The influence of chemical masking on Cas9 cleavage of target HBEGF DNA. Uncleaved HBEGF DNA (621 bp) cut to shorter cleavage fragments (311 bp and 310 bp) were indicated. Lane 1: DNA markers; lane 2: no Cas9 control; lane 3: original gRNA-HBEGF; lanes 4 - 6: gRNA-HBEGF with different masking levels.

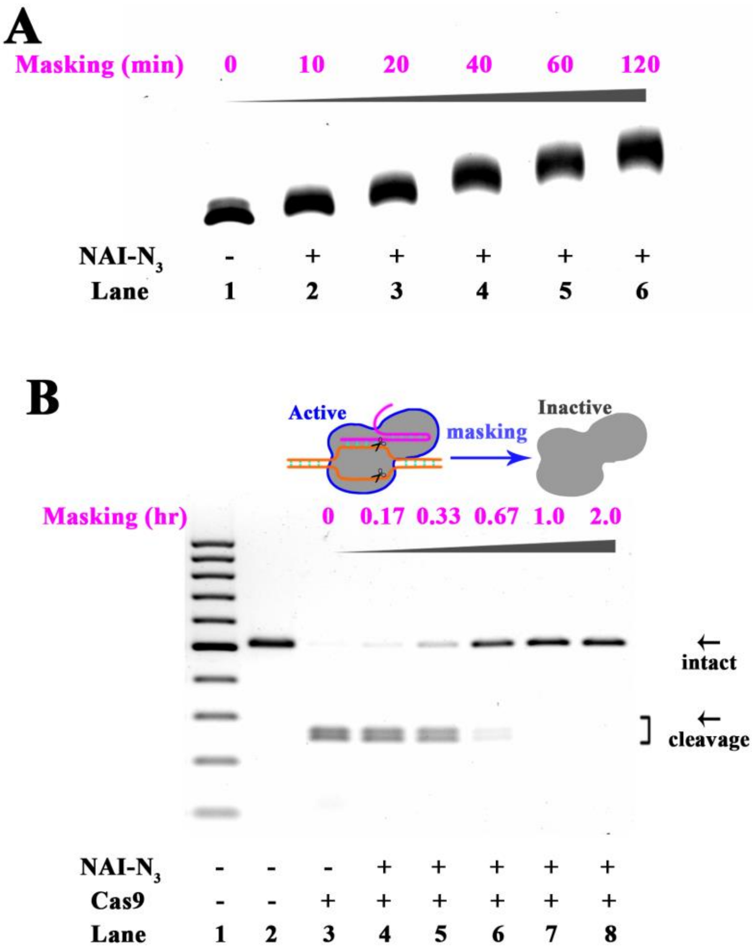

Supplementary Figure 22. **Conditional control of RNA-guided DNA cleavage.** Reactions were carried out as described in the Experimental Section. Image of representative data is shown here. (**A**) The gRNA (gRNA-ANTXR1) was synthesized by *in vitro* transcription with T7 RNA polymerase. After electrophoresis, the GelRed-stained gRNA-ANTXR1 was visualized using a Pharos FX Molecular imager (Bio-Rad, USA) in the fluorescence mode (λex = 590 nm). In this assay, the more slowly migrating bands represented the higher-molecular weight gRNAs with acylation. (**B**) The influence of chemical masking on Cas9 cleavage of target ANTXR1 DNA. Uncleaved ANTXR1 DNA (507 bp) cut to shorter cleavage fragments (264 bp and 243 bp) were indicated. Lane 1: DNA markers; lane 2: no Cas9 control; lane 3: original gRNA-ANTXR1; lanes 4 - 8: gRNA-ANTXR1 with different masking levels.

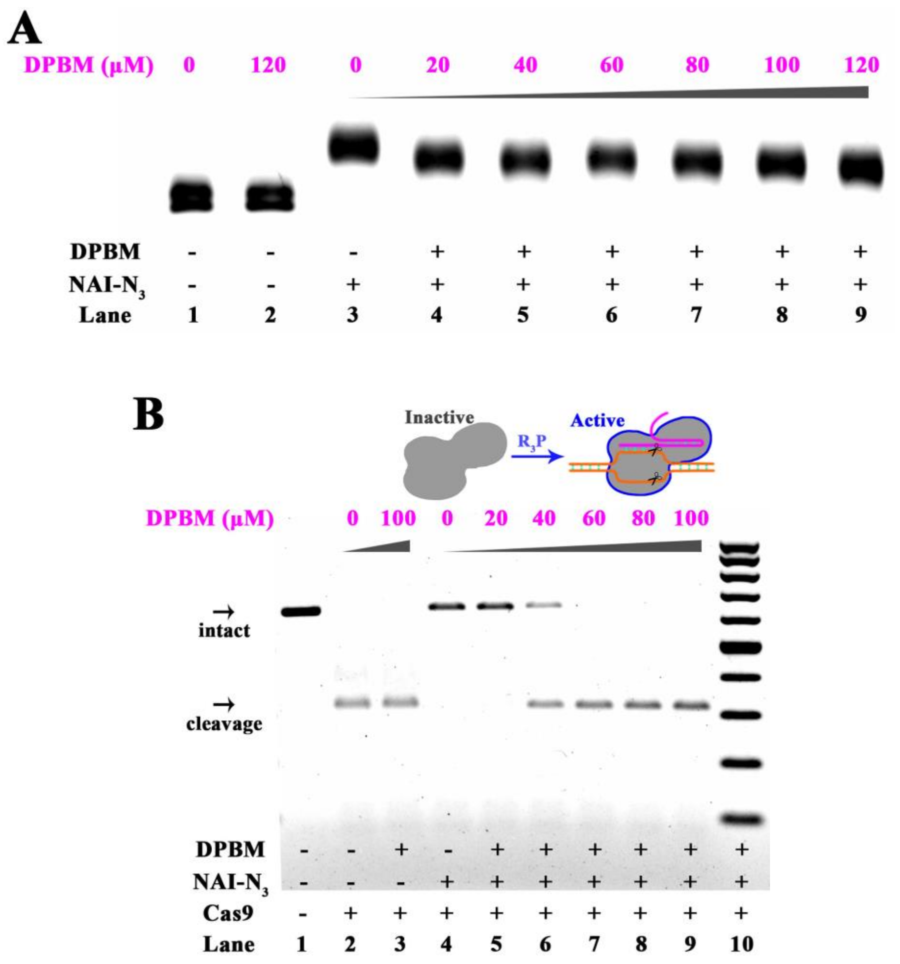

Supplementary Figure 23. **Conditional control of RNA-guided DNA cleavage.** Reactions were carried out as described in the Experimental Section. All samples were tested in three biological replicates. Image of representative data was shown here. (**A**) Staudinger reduction drives the removal of AMN groups with a concentration-dependent manner. The un-masking of acylated gRNA-HBEGF was confirmed by the appearance of faster-migrating bands, compared with the acylated sample. Lane 1: original gRNA-HBEGF; lane 2: original gRNA-HBEGF, 120 μM DPBM; lanes 3 - 9: acylated gRNA-HBEGF (200 mM NAI-N_3, 1.0 hr), different concentrations of DPBM. (**B**) The influence of DPBM on Cas9 cleavage. The CRISPR/Cas9 system with acylated gRNA-HBEGF (200 mM NAI-N_3, 1.0 hr) was treated with various concentrations of DPBM ranging from 0 to 100 μM. Uncleaved HBEGF DNA (621 bp) cut to shorter cleavage fragments (311 bp and 310 bp) were indicated. Lanes 1: no Cas9 control; lane 2: original gRNA-HBEGF; lane 3: original gRNA-HBEGF, 100 μM DPBM; lanes 4 - 9: acylated gRNA-HBEGF (200 mM NAI-N_3, 1.0 hr), different concentrations of DPBM; lane 10: DNA markers.

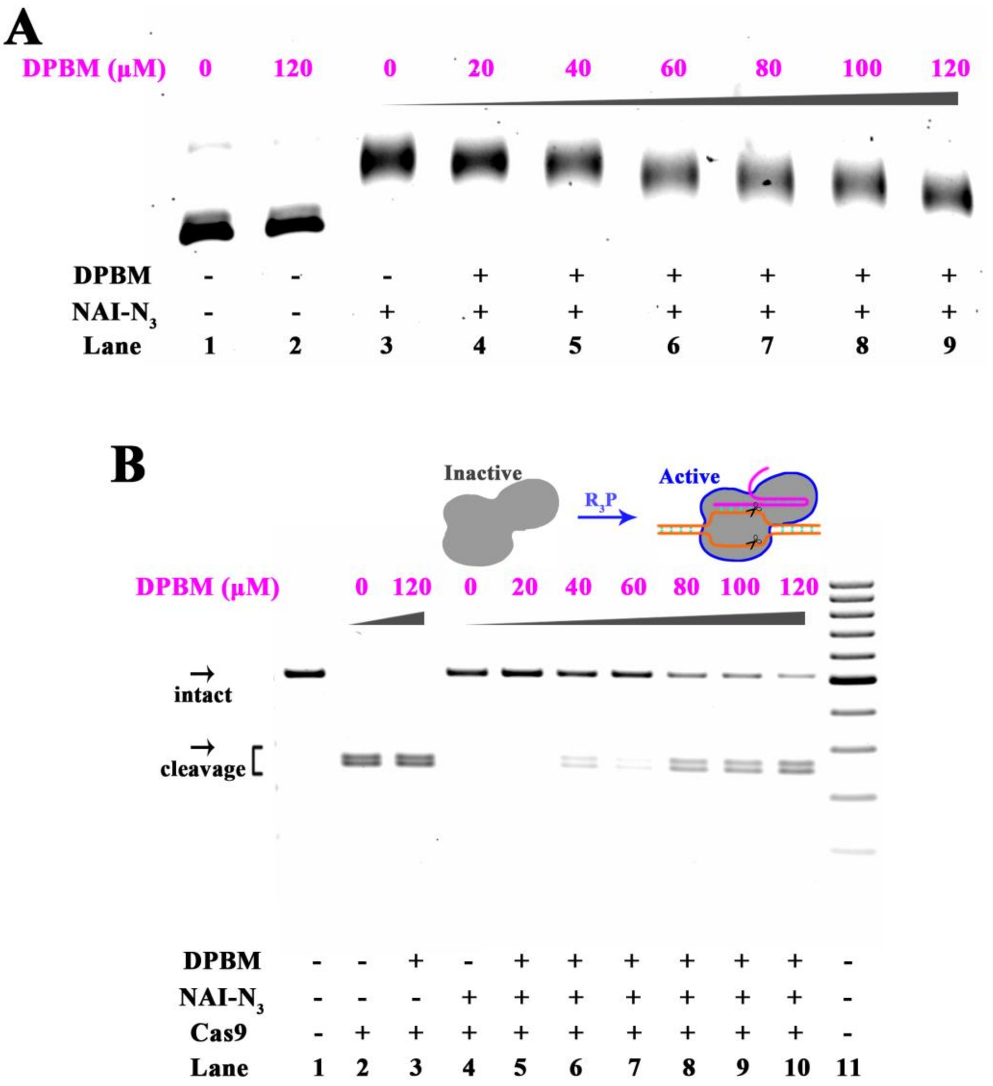

Supplementary Figure 24. **Conditional control of RNA-guided DNA cleavage.** Reactions were carried out as described in the Experimental Section. All samples were tested in three biological replicates. Image of representative data was shown here. (**A**) Staudinger reduction drives the removal of AMN groups with a concentration-dependent manner. The un-masking of acylated gRNA-ANTXR1 was confirmed by the appearance of faster-migrating bands, compared with the acylated sample. Lane 1: original gRNA-ANTXR1; lane 2: original gRNA-ANTXR1, 120 µM DPBM; lanes 3 - 9: acylated gRNA-ANTXR1 (200 mM NAI-N_3, 1.0 hr), different concentrations of DPBM. (**B**) The influence of DPBM on Cas9 cleavage. The CRISPR/Cas9 system with acylated gRNA-ANTXR1 (200 mM NAI-N_3, 1.0 hr) was treated with various concentrations of DPBM ranging from 0 to 120 µM. Uncleaved ANTXR1 DNA (507 bp) cut to shorter cleavage fragments (264 bp and 243 bp) were indicated. Lanes 1: no Cas9 control; lane 2: original gRNA-ANTXR1; lane 3: original gRNA-ANTXR1, 120 µM DPBM; lanes 4 - 10: acylated gRNA-ANTXR1 (200 mM NAI-N_3, 1.0 hr), different concentrations of DPBM; lane 11: DNA markers.

A

>gi|568815579|ref|NC_000019.10|:15862760-15863054 Homo sapiens chromosome 19, GRCh38.p12 Primary Assembly

TTCTCCATGTTCCGCTAGCACTAAAAAGACCCAAGAATCCCCCGGGCATTGGGCTGCC
TTTCCGAGATCCCTGCTACAGGAAGCCCCC**GTC**^**ACCAGTGCCGAAAGGA**GCAGAG
TCCCCGCCCTTCCCCCACCTCCCTTTCCCCACTCCTCACCATTGCGTGCCTGGACAACC
TTTATTTGCCTGAACATTTCACCCCGAATGAAGTCCCGGTCATGTCCGGTGTCTGCCA
GCCCCGGTGTCACTGAGAGACCCAAAGAGAACGCGCTGGTAAAGGTCTTGGCAGAGT
TGCGGGTT

B

>gi|568815596|ref|NC_000002.12|:3550774-3551255 Homo sapiens chromosome 2, GRCh38.p12 Primary Assembly

AGGACAATGGCGCTGTCTCCACGGCAGGGTCACCTTCACTCCCAGGGAGCCATCCTCC
AGAGACGAGCAGCGCCTCAGCATGACGGTGACCCTGCCTGCCAGAGGGCCCAGTCTC
CTGACCAGTGCTGGTCTAATTTCTTCCCTGGCATATTTGCTGCTAGTTAAGTGTGGTTC
CCGTCTTTGCTTCTTTCCCTCTAGATAGAGGGTATGATTGACACACAAAGGCTGTAAA
GAACAAGGTAAAAAAGGACTGACGGGGGCA**TTCTCAGGCCACTGGT**^**GAC**CAGGA
CACTTCAGTAGTGTGGTGGGATCAGAGGTCAGGTTGCACAGTGTTAAAAGAGCTGGG
CGAGAGGGCAAATAGAGGTGGTACGCGCAGGAAGTGGAAGTACTTGTCCGAAGGAC
GGCAGACTGAGCTCAGCAGTGGCGAAGGGGCAAAGATGGGGATGTGCTCCTCACAG
GAGCGGGGCTGAGAGCCAAGGGGAGTG

Supplementary Figure 25. Schematic illustration of two off-target sites known to be targeted by gRNA-HBEGF RNA. Off-target sites were chosen based on analysis using the CRISPR design tool (http://tools.genome-engineering.org). The target loci and PCR primer loci were indicated by blue color and underlining, respectively. Red caret showed the cleavage sites by Cas9 nuclease. PCR primers flanking the target regions were designed by Primer 3 software. We generated two different off-target targets (t-OT1-HBEGF and t-OT2-HBEGF) carrying the target loci from HeLa-OC genomic DNA. (A) The t-OT1-HBEGF was demonstrated. (B) The t-OT2-HBEGF was demonstrated.

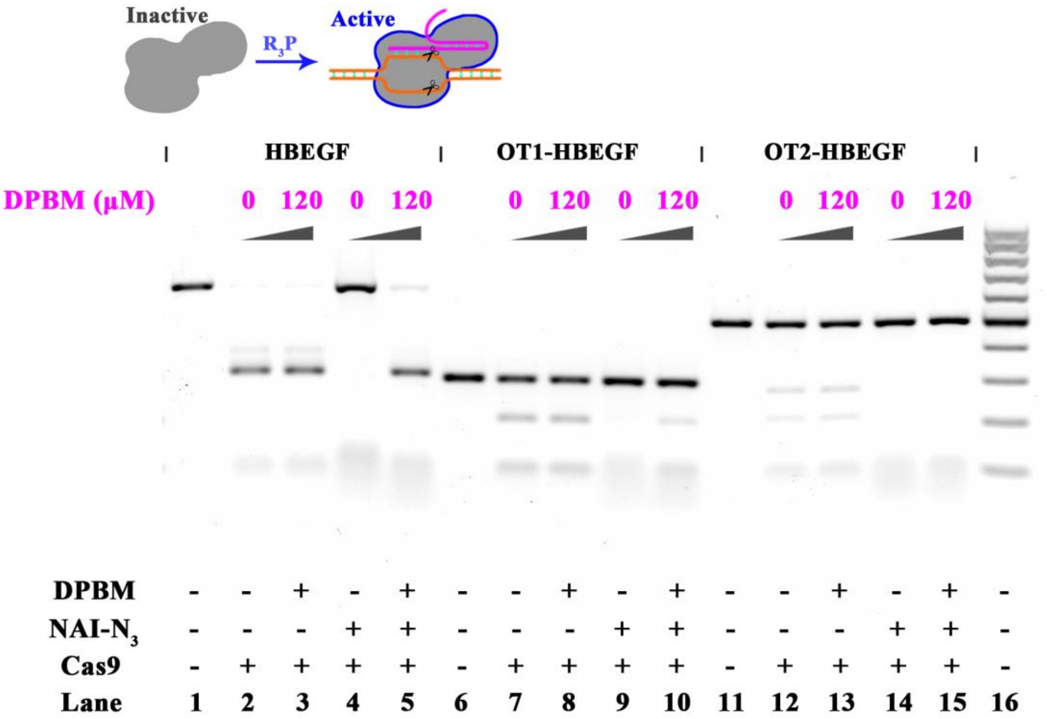

Supplementary Figure 26. **The cleavage of two corresponding off-target sites known to be targeted by the HBEGF RNA.** Reactions were carried out as described in the Experimental Section. Image of representative data was shown here. Lane 1: no Cas9 control; lane 2: original gRNA-HBEGF; lane 3: original gRNA-HBEGF, 120 μM DPBM; lane 4: acylated gRNA-HBEGF (200 mM NAI-N_3, 1.0 hr); lane 5: acylated gRNA-HBEGF (200 mM NAI-N_3, 1.0 hr), 120 μM DPBM; lane 6: no Cas9 control; lane 7: original gRNA-HBEGF; lane 8: original gRNA-HBEGF, 120 μM DPBM; lane 9: acylated gRNA-HBEGF (200 mM NAI-N_3, 1.0 hr); lane 10: acylated gRNA-HBEGF (200 mM NAI-N_3, 1.0 hr), 120 μM DPBM; lane 11: no Cas9 control; lane 12: original gRNA-HBEGF; lane 13: original gRNA-HBEGF, 120 μM DPBM; lane 14: acylated gRNA-HBEGF (200 mM NAI-N_3, 1.0 hr); lane 15: acylated gRNA-HBEGF (200 mM NAI-N_3, 1.0 hr), 120 μM DPBM; lane 16: DNA markers. Lanes 1-5: Uncleaved HBEGF DNA (621 bp) cut to shorter cleavage fragments (311 bp and 310 bp) were indicated. (**A**) Uncleaved t-OT1-HBEGF (295 bp) cut to shorter cleavage fragments (204 bp and 91 bp) were indicated. (B) Uncleaved t-OT2-HBEGF (482 bp) cut to shorter cleavage fragments (278 bp and 204 bp) were indicated.

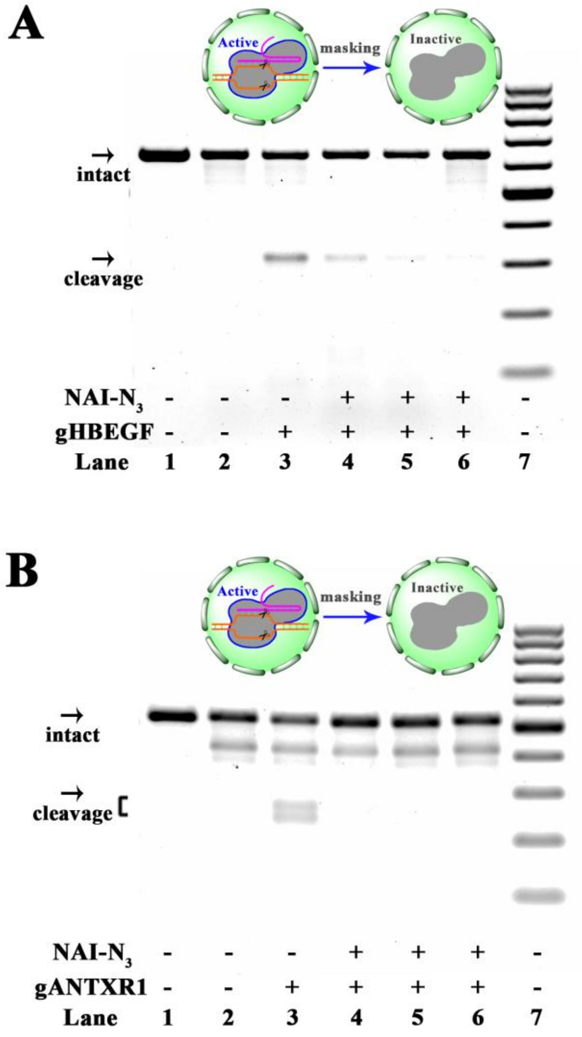

Supplementary Figure 27. **Gene editing in live cells.** The T7E1 nuclease assay was performed 24 hr post-transfection using Cas9-expressing HeLa-OC cells transfected with gRNA-HBEGF or gRNA-ANTXR1 with different treatments. All samples were tested in three biological replicates. Image of representative data was shown here. (**A**) Uncleaved HBEGF DNA (621 bp) cut to shorter cleavage fragments (311 bp and 310 bp) were indicated. Lane 1: target control; lane 2: no gRNA-HBEGF control; lane 3: original gRNA-HBEGF; lane 4: acylated gRNA-HBEGF (200 mM NAI-N_3, 20 min); lane 5: acylated gRNA-HBEGF (200 mM NAI-N_3, 40 min); lane 6: acylated gRNA-HBEGF (200 mM NAI-N_3, 1.0 hr); lane 7: DNA markers. (**B**) Uncleaved ANTXR1 DNA (507 bp) cut to shorter cleavage fragments (264 bp and 243 bp) were indicated. Lane 1: target control; lane 2: no gRNA-ANTXR1 control; lane 3: original gRNA-ANTXR1; lane 4: acylated gRNA-ANTXR1 (200 mM NAI-N_3, 20 min); lane 5: acylated gRNA-ANTXR1 (200 mM NAI-N_3, 40 min); lane 6: acylated gRNA-ANTXR1 (200 mM NAI-N_3, 1.0 hr); lane 7: DNA markers.

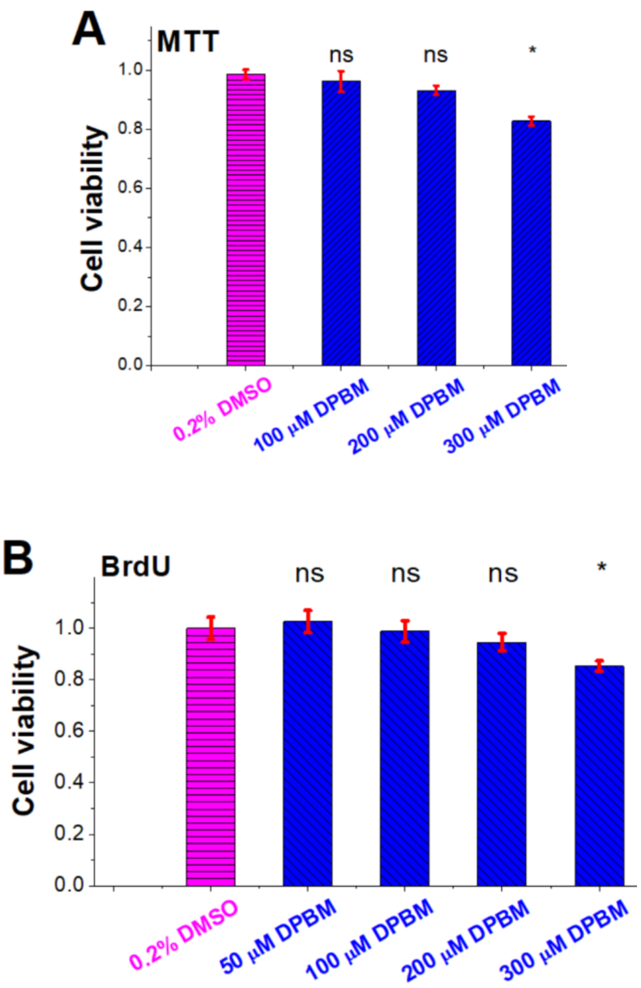

Supplementary Figure 28. **The cytotoxicity assay.** (**A**) An MTT assay assay was performed as described in the Experimental Section. (**B**) A BrdU was performed as described in the Experimental Section. HeLa-OC cells were treated for 24 hr with DPBM, and the effect on cell growth was analyzed. Values were plotted relative to the mean of DMEM control set to 100% (= relative growth). All data were presented as the means ± SEM from three independent experiments. The cytotoxicity threshold for DPBM was higher than 200 µM. Error bars: ± SEM. P-values: * P < 0.05 against the 0.2% DMSO treatment (Student's t-test), ns. - not significant against the 0.2% DMSO treatment.

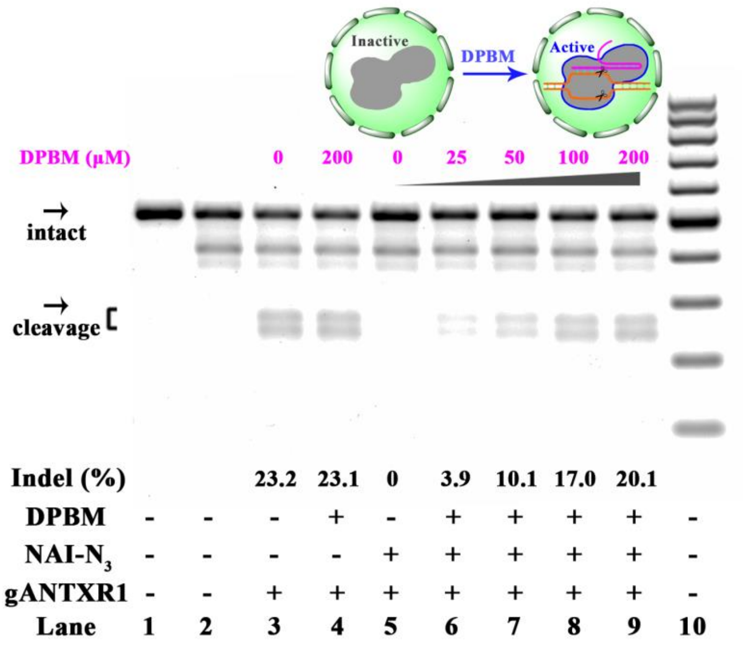

Supplementary Figure 29. **Gene editing in live cells.** The T7E1 nuclease assay was performed using Cas9-expressing HeLa-OC cells transfected with gRNA-ANTXR1 with different treatments. Uncleaved ANTXR1 DNA (507 bp) cut to shorter cleavage fragments (264 bp and 243 bp) were indicated. All samples were tested in three biological replicates. Image of representative data was shown here. Lane 1: target control; lane 2: no gRNA-ANTXR1 control; lane 3: original gRNA-ANTXR1; lane 4: original gRNA-ANTXR1, 200 μM DPBM; lane 5: acylated gRNA-ANTXR1 (200 mM NAI-N_3, 1.0 hr); lanes 6-9: acylated gRNA-ANTXR1 (200 mM NAI-N_3, 1.0 hr), different concentrations of DPBM; lane 10: DNA markers.

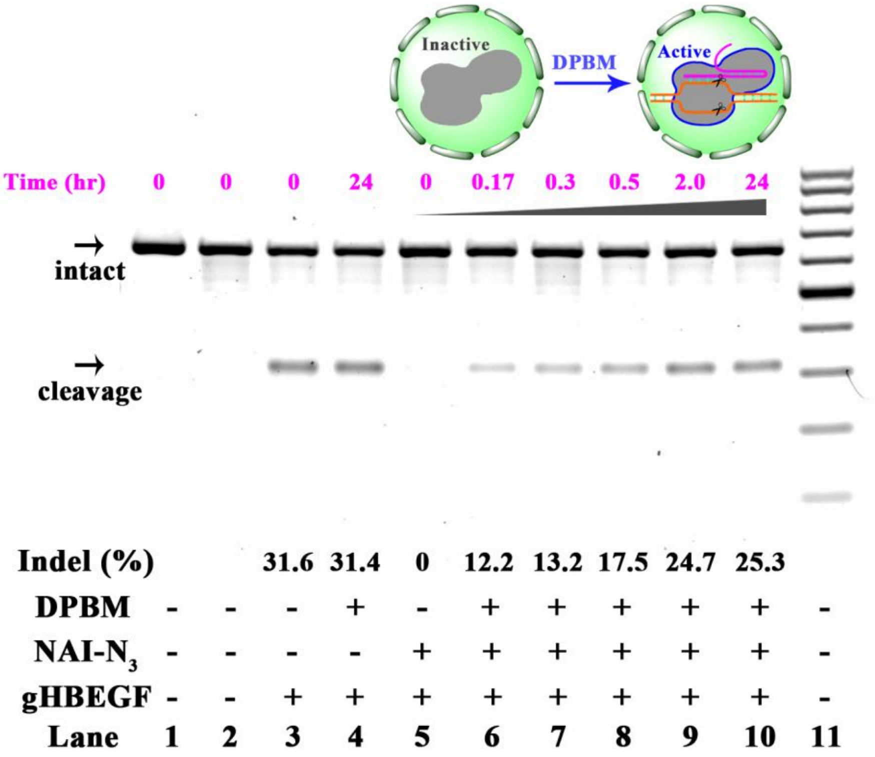

Supplementary Figure 30. **The kinetics of in cell Staudinger reaction.** The T7E1 nuclease assay was performed using Cas9-expressing HeLa-OC cells transfected with gRNA-HBEGF with different treatments. Uncleaved HBEGF DNA (621 bp) cut to shorter cleavage fragments (311 bp and 310 bp) were indicated. All samples were tested in three biological replicates. Image of representative data was shown here. Lane 1: target control; lane 2: no gRNA-HBEGF control; lane 3: original gRNA-HBEGF; lane 4: original gRNA-HBEGF, 200 μM DPBM; lane 5: acylated gRNA-HBEGF (200 mM NAI-N_3, 1.0 hr); lanes 6-10: acylated gRNA-HBEGF (200 mM NAI-N_3, 1.0 hr), the 200 μM DPBM treatment with different durations; lane 11: DNA markers.

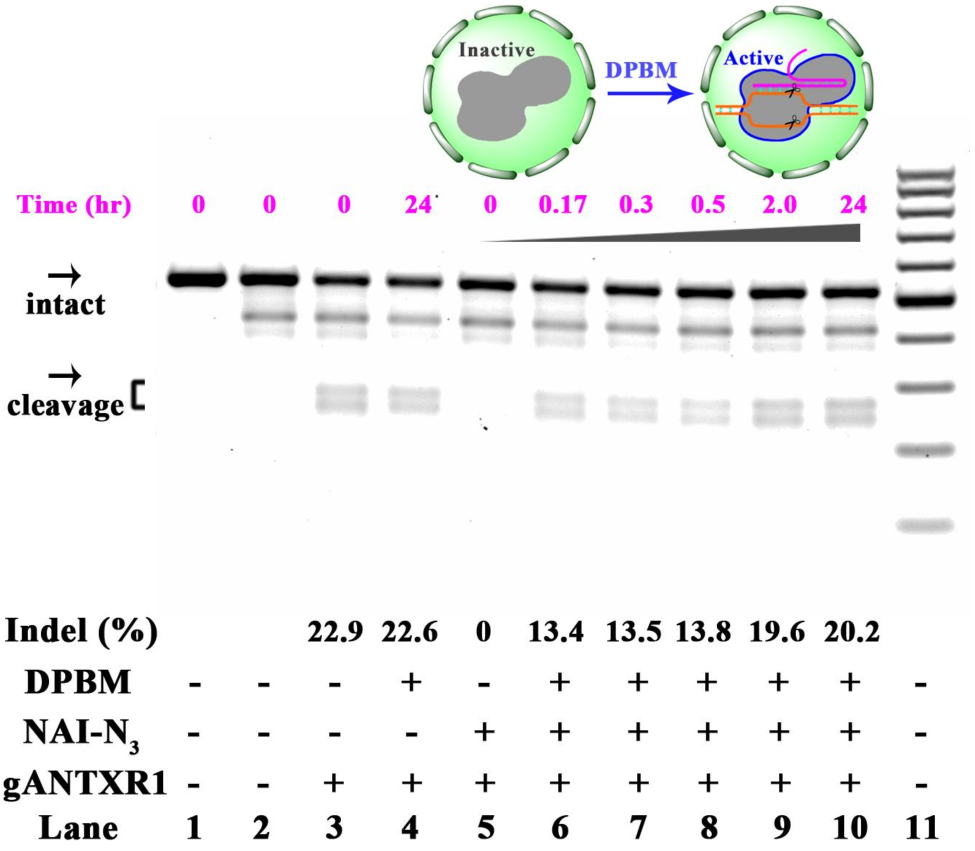

Supplementary Figure 31. **The kinetics of in cell Staudinger reaction.** The T7E1 nuclease assay was performed using Cas9-expressing HeLa-OC cells transfected with gRNA-ANTXR1 with different treatments. Uncleaved ANTXR1 DNA (507 bp) cut to shorter cleavage fragments (264 bp and 243 bp) were indicated. All samples were tested in three biological replicates. Image of representative data was shown here. Lane 1: target control; lane 2: no gRNA-ANTXR1 control; lane 3: original gRNA-ANTXR1; lane 4: original gRNA-ANTXR1, 200 μM DPBM; lane 5: acylated gRNA-ANTXR1 (200 mM NAI-N_3, 1.0 hr); lanes 6-10: acylated gRNA-ANTXR1 (200 mM NAI-N_3, 1.0 hr), the 200 μM DPBM treatment with different durations; lane 11: DNA markers.

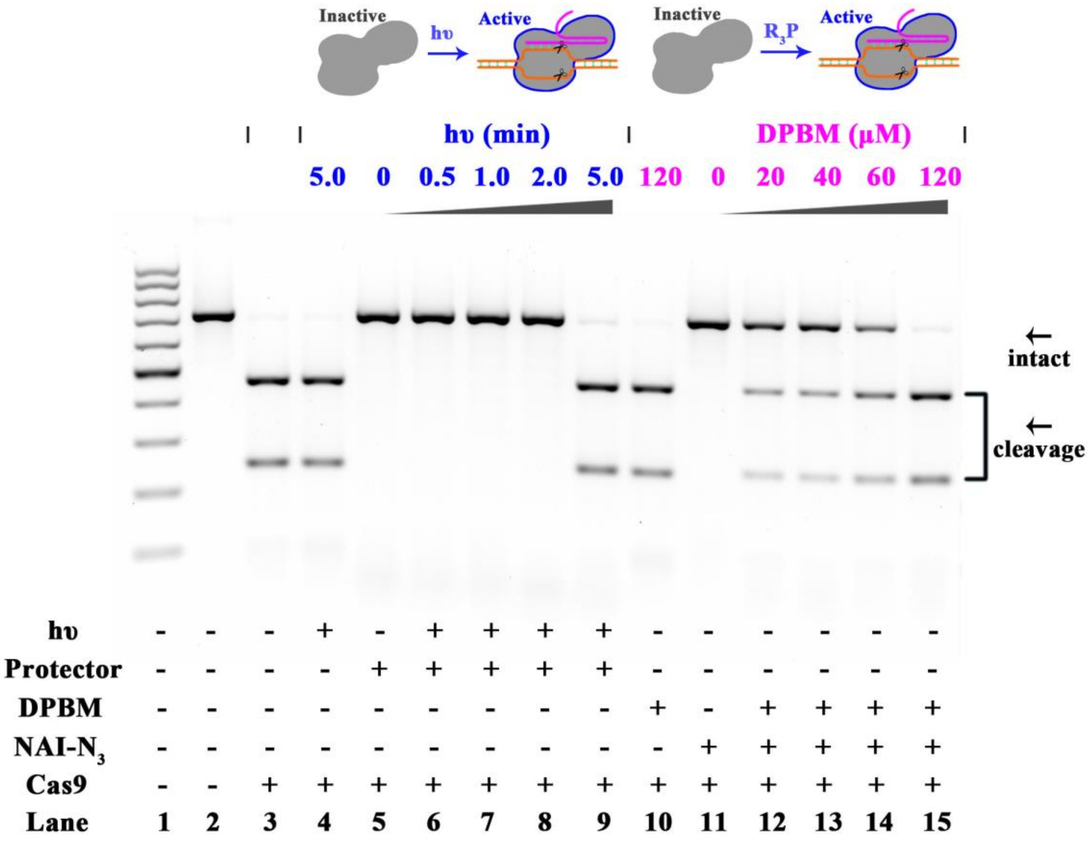

Supplementary Figure 32. **Conditional control of RNA-guided DNA cleavage.** Reactions were carried out as described in the Experimental Section. The t-GFP1 was used as the double-stranded DNA substrate for Cas9 cleavage. Uncleaved t-GFP1 DNA (702 bp) cut to shorter cleavage fragments (469 bp and 233 bp) were indicated. All samples were tested in three biological replicates. Representative gel electrophoresis data from the *in vitro* cleavage assay was shown here. The CRISPR-plus system with a 1:2 ratio of gRNA-GFP/protector DNA (Protector-GFP) was treated with various periods of 365-nm light ranging from 0 to 5.0 min. The CRISPR/Cas9 system with acylated gRNA-GFP (200 mM NAI-N_3, 1.0 hr) was treated with various concentrations of DPBM ranging from 0 to 120 μM. Lane 1: DNA markers; lane 2: no Cas9 control; lane 3: original gRNA-GFP; lane 4: original gRNA-GFP, 5 min 365-nm light irradiation; lane 5: original gRNA-GFP/protector; lanes 6 - 9: original gRNA-GFP/protector, different periods of 365-nm light irradiation; lane 10: original gRNA-GFP, 120 μM DPBM; lane 11: acylated gRNA-GFP (200 mM NAI-N_3, 1.0 hr); lanes 12 - 15: acylated gRNA-GFP (200 mM NAI-N_3, 1.0 hr), different concentrations of DPBM.

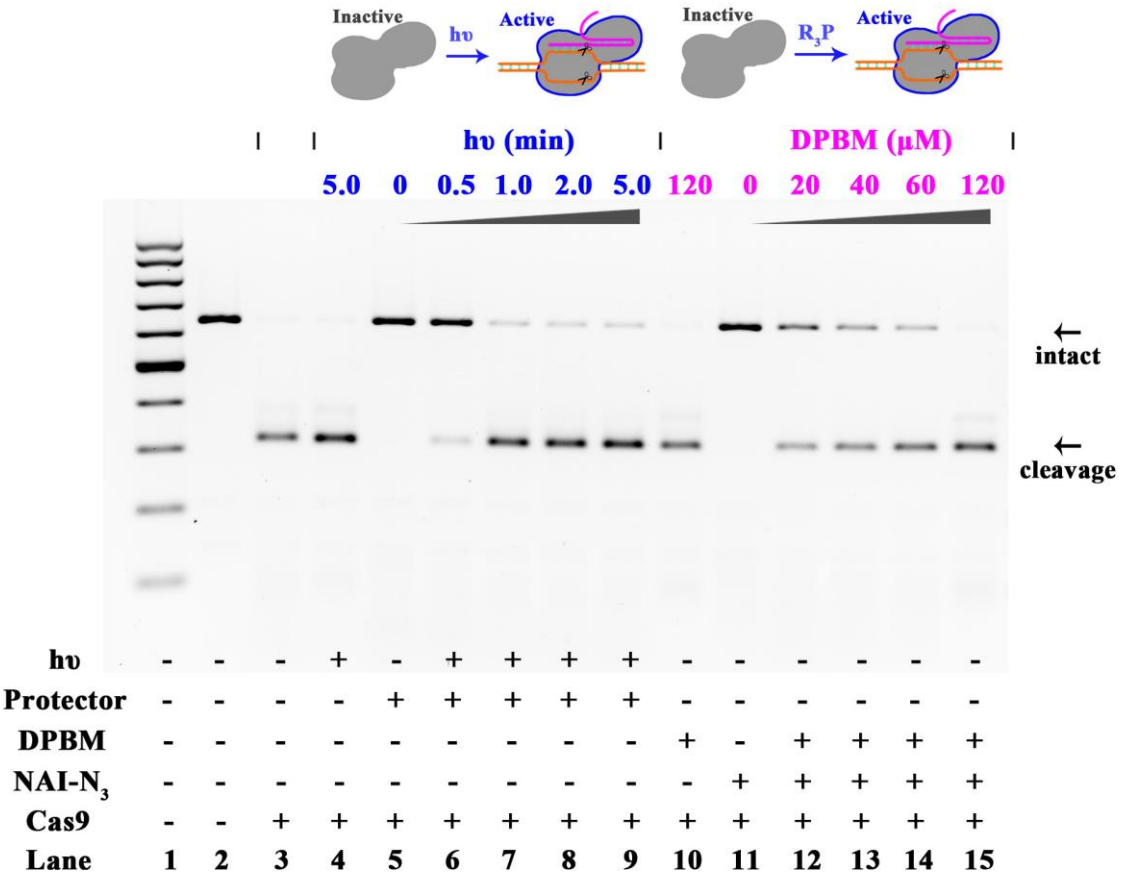

Supplementary Figure 33. **Conditional control of RNA-guided DNA cleavage.** Reactions were carried out as described in the Experimental Section. The t-HBEGF was used as the double-stranded DNA substrate for Cas9 cleavage. Uncleaved HBEGF DNA (621 bp) cut to shorter cleavage fragments (311 bp and 310 bp) were indicated. All samples were tested in three biological replicates. Representative gel electrophoresis data from the *in vitro* cleavage assay was shown here. The CRISPR-plus system with a 1:2 ratio of gRNA-HBEGF/protector DNA (Protector-HBEGF) was treated with various periods of 365-nm light ranging from 0 to 5.0 min. The CRISPR/Cas9 system with acylated gRNA-HBEGF (200 mM NAI-N_3, 1.0 hr) was treated with various concentrations of DPBM ranging from 0 to 120 μM. Lane 1: DNA markers; lane 2: no Cas9 control; lane 3: original gRNA-HBEGF; lane 4: original gRNA-HBEGF, 5 min 365-nm light irradiation; lane 5: original gRNA-HBEGF/protector; lanes 6 - 9: original gRNA-HBEGF/protector, different periods of 365-nm light irradiation; lane 10: original gRNA-HBEGF, 120 μM DPBM; lane 11: acylated gRNA-HBEGF (200 mM NAI-N_3, 1.0 hr); lanes 12 - 15: acylated gRNA-HBEGF (200 mM NAI-N_3, 1.0 hr), different concentrations of DPBM.

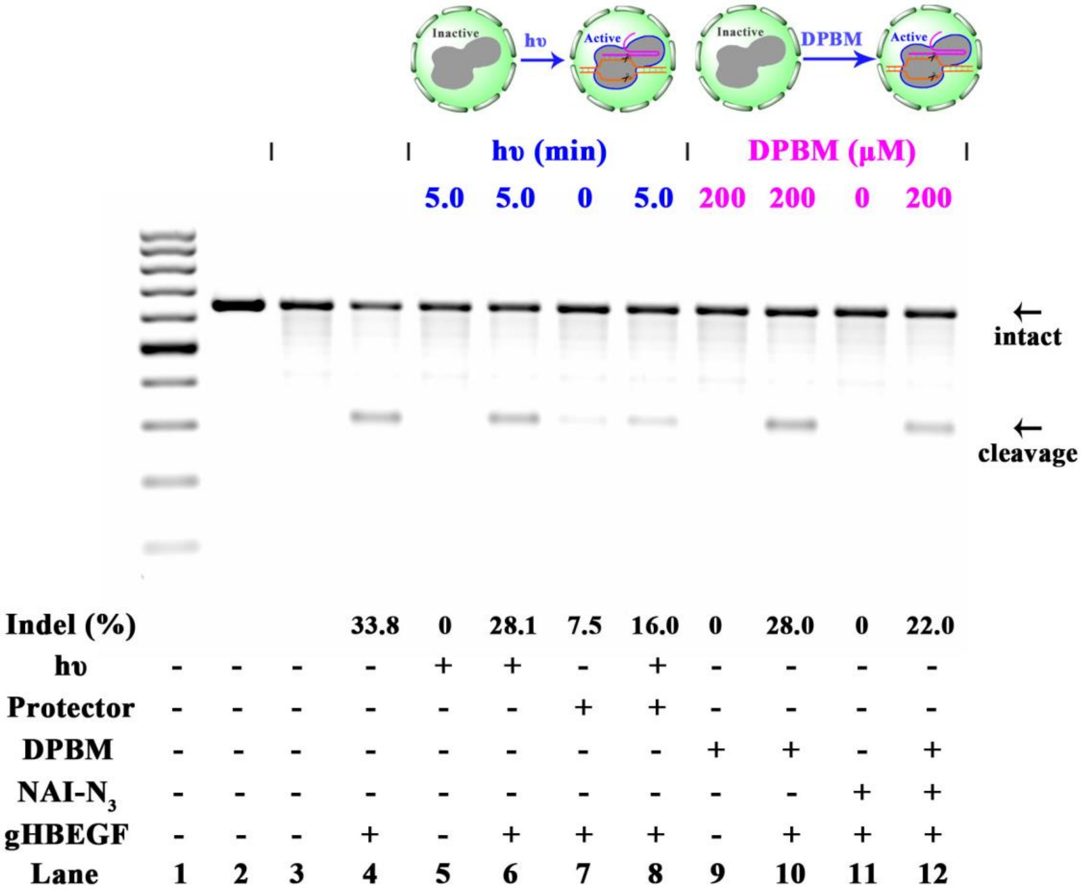

Supplementary Figure 34. **Gene editing in live cells.** The T7E1 nuclease assay was performed 24 hr post-transfection using Cas9-expressing HeLa-OC cells transfected with gRNA-HBEGF (or gRNA-HBEGF/Protector-HBEGF at a 1:2 ratio) with different treatments. Reactions were carried out as described in the Experimental Section. Uncleaved HBEGF DNA (621 bp) cut to shorter cleavage fragments (311 bp and 310 bp) were indicated. All samples were tested in three biological replicates. Representative gel electrophoresis data was shown here. Lane 1: DNA markers; lane 2: target control; lane 3: no gRNA-HBEGF control; lane 4: original gRNA-HBEGF; lane 5: no gRNA-HBEGF, 5 min 365-nm light irradiation; lane 6: original gRNA-HBEGF, 5 min 365-nm light irradiation; lane 7: original gRNA-HBEGF/protector; lanes 8: original gRNA-HBEGF/protector, 5 min 365-nm light irradiation; lane 9: no gRNA-HBEGF, 200 μM DPBM; lane 10: original gRNA-HBEGF, 200 μM DPBM; lane 11: acylated gRNA-HBEGF (200 mM NAI-N_3, 1.0 hr); lane 12: acylated gRNA-HBEGF (200 mM NAI-N_3, 1.0 hr), 200 μM DPBM.

Supplementary References:

1. Spitale, R.C., *et al.* Structural imprints in vivo decode RNA regulatory mechanisms. *Nature* **519**, 486-490 (2015).
2. Saneyoshi, H., *et al.* Triphenylphosphinecarboxamide: an effective reagent for the reduction of azides and its application to nucleic acid detection. *Org Lett* **16**, 30-33 (2014).
3. Deng, W., Shi, X., Tjian, R., Lionnet, T. & Singer, R.H. CASFISH: CRISPR/Cas9-mediated in situ labeling of genomic loci in fixed cells. *Proc Natl Acad Sci U S A* **112**, 11870-11875 (2015).
4. Jain, P.K., *et al.* Development of Light-Activated CRISPR Using Guide RNAs with Photocleavable Protectors. *Angew Chem Int Ed Engl* **55**, 12440-12444 (2016).
5. Polstein, L.R. & Gersbach, C.A. A light-inducible CRISPR-Cas9 system for control of endogenous gene activation. *Nat Chem Biol* **11**, 198-200 (2015).
6. Hemphill, J., Borchardt, E.K., Brown, K., Asokan, A. & Deiters, A. Optical Control of CRISPR/Cas9 Gene Editing. *J Am Chem Soc* **137**, 5642-5645 (2015).
7. Zetsche, B., Volz, S.E. & Zhang, F. A split-Cas9 architecture for inducible genome editing and transcription modulation. *Nat Biotechnol* **33**, 139-142 (2015).
8. Luo, J., Liu, Q., Morihiro, K. & Deiters, A. Small-molecule control of protein function through Staudinger reduction. *Nat Chem* **8**, 1027-1034 (2016).
9. Dow, L.E., *et al.* Inducible in vivo genome editing with CRISPR-Cas9. *Nat Biotechnol* **33**, 390-394 (2015).
10. Gonzalez, F., *et al.* An iCRISPR platform for rapid, multiplexable, and inducible genome editing in human pluripotent stem cells. *Cell Stem Cell* **15**, 215-226 (2014).
11. Chen, Y., *et al.* Engineering Human Stem Cell Lines with Inducible Gene Knockout using CRISPR/Cas9. *Cell Stem Cell* **17**, 233-244 (2015).
12. Davis, K.M., Pattanayak, V., Thompson, D.B., Zuris, J.A. & Liu, D.R. Small molecule-triggered Cas9 protein with improved genome-editing specificity. *Nat Chem Biol* **11**, 316-318 (2015).
13. Pan, Y., *et al.* Near-infrared upconversion-activated CRISPR-Cas9 system: A remote-controlled gene editing platform. *Sci Adv* **5**, eaav7199 (2019).
